# Single Diode Solar Cells—Improved Model and Exact Current–Voltage Analytical Solution Based on Lambert’s W Function

**DOI:** 10.3390/s22114173

**Published:** 2022-05-31

**Authors:** Muhyaddin Rawa, Martin Calasan, Abdullah Abusorrah, Abdullah Ali Alhussainy, Yusuf Al-Turki, Ziad M. Ali, Hatem Sindi, Saad Mekhilef, Shady H. E. Abdel Aleem, Hussain Bassi

**Affiliations:** 1 Smart Grids Research Group, Center of Research Excellence in Renewable Energy and Power Systems, King Abdulaziz University, Jeddah 21589, Saudi Arabia; mrawa@kau.edu.sa (M.R.); aabusorrah@kau.edu.sa (A.A.); ahalhussainy0001@stu.kau.edu.sa (A.A.A.); yaturki@kau.edu.sa (Y.A.-T.); hfsindi@kau.edu.sa (H.S.); saad@um.edu.my (S.M.); hussain.bassi@moenergy.gov.sa (H.B.); 2 Department of Electrical and Computer Engineering, Faculty of Engineering, K. A. CARE Energy Research and Innovation Center, Jeddah 21589, Saudi Arabia; 3 Faculty of Electrical Engineering, University of Montenegro, 81000 Podgorica, Montenegro; martinc@ucg.ac.me; 4 Electrical Engineering Department, College of Engineering, Prince Sattam bin Abdulaziz University, Wadi Addawaser 11991, Saudi Arabia; 5 Electrical Engineering Department, Aswan Faculty of Engineering, Aswan University, Aswan 81542, Egypt; 6 Power Electronics and Renewable Energy Research Laboratory (PEARL), Department of Electrical Engineering, University of Malaya, Kuala Lumpur 50603, Malaysia; 7 Department of Electrical Engineering, Valley High Institute of Engineering and Technology, Science Valley Academy, Qalyubia 44971, Egypt; shady.sebai@sva.edu.eg; 8 Ministry of Energy, Riyadh 11191, Saudi Arabia

**Keywords:** Lambert’s W function, mathematical models, optimization, parameter estimation, photovoltaics, solar cells, special trans function theory

## Abstract

There are three standard equivalent circuit models of solar cells in the literature—single-diode, double-diode, and triple-diode models. In this paper, first, a modified version of the single diode model, called the Improved Single Diode Model (ISDM), is presented. This modification is realized by adding resistance in series with the diode to enable better power loss dissipation representation. Second, the mathematical expression for the current–voltage relation of this circuit is derived in terms of Lambert’s W function and solved by using the special trans function theory. Third, a novel hybrid algorithm for solar cell parameters estimation is proposed. The proposed algorithm, called SA-MRFO, is used for the parameter estimation of the standard single diode and improved single diode models. The proposed model’s accuracy and the proposed algorithm’s efficiency are tested on a standard RTC France solar cell and SOLAREX module MSX 60. Furthermore, the experimental verification of the proposed circuit and the proposed solar cell parameter estimation algorithm on a solar laboratory module is also realized. Based on all the results obtained, it is shown that the proposed circuit significantly improves current–voltage solar cell representation in comparison with the standard single diode model and many results in the literature on the double diode and triple diode models. Additionally, it is shown that the proposed algorithm is effective and outperforms many literature algorithms in terms of accuracy and convergence speed.

## 1. Introduction

The modeling, planning, management, and optimal operation of solar energy systems require knowledge of accurate models of the components used [1,2], which relies on the accurate modeling of the equivalent circuits of solar cells and panels [3]. The accuracy of a solar photovoltaic (PV) model greatly influences system design [4]. In this regard, there are three equivalent circuit models of solar cells widely used in the available literature. The first and most widely accepted model is the single-diode solar cell model (SDM) [5,6,7]. This five-parameter SDM is prevalent in the literature due to its simplicity. Besides, the seven-parameter double-diode model (DDM) [8,9] and nine-parameter triple-diode solar cell model (TDM) [10] make use of additional diodes in their models to describe the physical nature of solar cells. Although these models provide good accuracy in modeling solar cells, they have a more complex structure since they are represented with more parameters [11,12].

Several solar cell parameter estimation approaches have been developed in scientific publications [13,14,15,16]. For instance, it is possible to estimate the parameters of solar cells from nameplate data, i.e., using the catalogue data of the manufacturer [13,17]. However, different research works have shown that this approach has drawbacks because real-world conditions differ from the operating conditions assumed when these cells were tested in factories. Additionally, it is expected to have incomplete data or missing parameters in data sheets provided by manufacturers. Thus, it is preferable to find these missing parameters based on the measured voltage–current characteristics of these cells [14,18]. Unfortunately, regardless of the approach or the solar cell model used, solar cells are characterized by the nonlinearity of the mathematical relation of currents and voltages. This means that estimating the parameters is associated with solving high nonlinear equations [19].

Afterward, several approaches have been proposed in the literature for estimating the precise parameters of diode models of solar PV equivalent circuits. The first approach relies on applying numerical methods to estimate the values of these parameters, but this approach is time-consuming [15]. Additionally, these approaches are based on iterative techniques, and it is well known that the performance of iterative techniques is highly dependent on the initial values provided by the programmer/designer. Added to that, they may suffer from local solutions problems. The second method is based on solving the equations analytically [14]. However, this approach necessitates several approximations/relaxations as the mathematical relation between currents and voltages is nonlinear, affecting the model’s accuracy. The most widely accepted methods in this research point are based on the application of metaheuristic algorithms [20,21]. Metaheuristic algorithms are characterized by the simplicity of application and independence on the initial values of the unknown parameters. Today, over 100 different algorithms can be found to estimate solar cell parameters. Generally, they can be categorized into several groups (All acronyms of algorithms are explained in a list of abbreviations):

- Bio-inspired algorithms (BIA) mimic ideas, processes, or biological behaviors in nature. The main representatives are MADE [22], ISCE [23], BPFPA [8], GAMNU [24], and GA [25].
- Swarming-based algorithms (SBA) mimic swarming behaviors of birds, cats, bees, fish, or others. The main representatives are EHHO [26], CPMPSO [27], FPSO [28], MPSO [29], FA [30], MSSO [31], CSO [32], ABC [25,31,33], WHHO [21], and PSO [33].
- Physics- and chemistry-based algorithms (P-CBA) mimic physical or chemical ideas or concepts of estimation procedures. The prominent representatives are ER-WCA [34,35], WDO [36], and HS [35].
- Teaching- and learning-based algorithms (T-LBA) mimic the teaching process with students and schoolchildren. The main representatives are GOTLBO [12], STLBO [12,37], SATLBO [38], GSK [39], EOTLBO [40], and LETLBO [9].
- Chaotic-based algorithms (CBA) mimic chaotic processes from science and nature. The main representatives are ILCOA [41], COA [10,35,42], CWOA [41], CNMSMA [4], and CLSHADE [10].
- Mathematical-based algorithms (MBA) use mathematical expressions and equations for some process descriptions. The leading representative is ISCA [43].
- Hybrid algorithms (HA) combine different analytical and numerical optimization methods, and so on. The main representatives are BHCS [44], HFAPS [30], and TLABC [9,45].

Predominantly, most of the research works are oriented toward the proposal of new algorithms to estimate parameters of the solar diode models. At the same time, most of them use some of the solar cell models and test them on standard solar cells, such as RTC France [9,46,47,48], Solarex MSX 60 [10,17,35], or others, or perform experimental verification on real cells [10]. Their primary focus is the comparisons of algorithms in terms of the speed of convergence required in a certain number of iterations, time per iteration, statistical measures, and so on [35]. It is clear that this research point can be further expanded by developing new models of solar cells. Consequently, this paper addresses this research point.

In this work, we propose a new simple six-parameter diode model of solar cells that will not further complicate the model, but will increase the accuracy of the estimation of solar cell parameters, i.e., improve the accuracy of modeling current–voltage characteristics. Namely, an improved single diode model (ISDM) is proposed in this work, including an additional resistor that models the losses during solar energy conversion into electricity. The mathematical expression of the current–voltage characteristic of the proposed model was derived, in which the derived equation is highly nonlinear (transcendental type). An analytical solution to the current as a function of the voltage is proposed in terms of Lambert’s W function and is further solved by using the special trans function theory (STFT). Additionally, investigating the accuracy of the proposed model was performed on several solar cells and modules. Note that different models of solar cells are listed in [16], which deals with equivalent models for solar cells in which the resistance of the diode is included in two-diode and three-diode models of solar cells. However, in [16], no analytical expressions for current–voltage dependence are given, nor is the solution of the same analyzed. Therefore, this work represents a forward step in terms of developing a new one-diode solar cell model and its mathematical explanation.

Besides, a novel hybrid algorithm for solar cell parameters estimation is proposed. The proposed algorithm, called SA-MRFO, is based on simulated annealing (SA) and Manta ray foraging optimization (MRFO), in which the SA algorithm is used to initialize the population of the MRFO, and it is used for parameters estimation of the standard and improved single-diode models. The proposed algorithm results are compared with those obtained by other algorithms presented in the literature to validate their effectiveness and accuracy. Moreover, for the RTC France solar cell, a comparison of the results with the corresponding ones obtained by applying deterministic methods was carried out.

Therefore, the main contributions of this work are outlined as follows:

- A new original single-diode solar cell model is proposed.
- The mathematical expression of the current–voltage characteristic of the proposed model is derived.
- The accuracy of the proposed model is tested, and its advantages over the single-diode model are shown.
- The accuracy of the proposed model is compared with the precision of two-diode and three-diode models, and it is shown that the results obtained are even better than some literature-known solutions of these models.
- The experimental verification of the proposed circuit and the proposed solar cell parameter estimation algorithm on a solar laboratory module is made, and the applicability of the proposed model is demonstrated.
- The advantage of applying the proposed algorithm compared with different algorithms in the literature is shown in terms of convergence rate, standard deviation, and Wilcoxon rank-sum test.

The rest of the paper is arranged as follows. The common diode models of solar PV equivalent circuits are presented in Section 2. The analytical formulation of the new six-parameter solar cell model—ISDM—is presented in Section 3. The proposed simulated annealing–Manta ray foraging optimization is presented in Section 4. In Section 5, the numerical outcomes and findings for two types of solar cells are presented, analyzed, and discussed. The experimental verification of the proposed model was made on measured data from a solar laboratory module, and the applicability of the proposed model is demonstrated in Section 6. Finally, the conclusions, study limitations, and future works are given in Section 7.

## 2. Common Diode Models of Solar PV Equivalent Circuits

**Figure 1:**
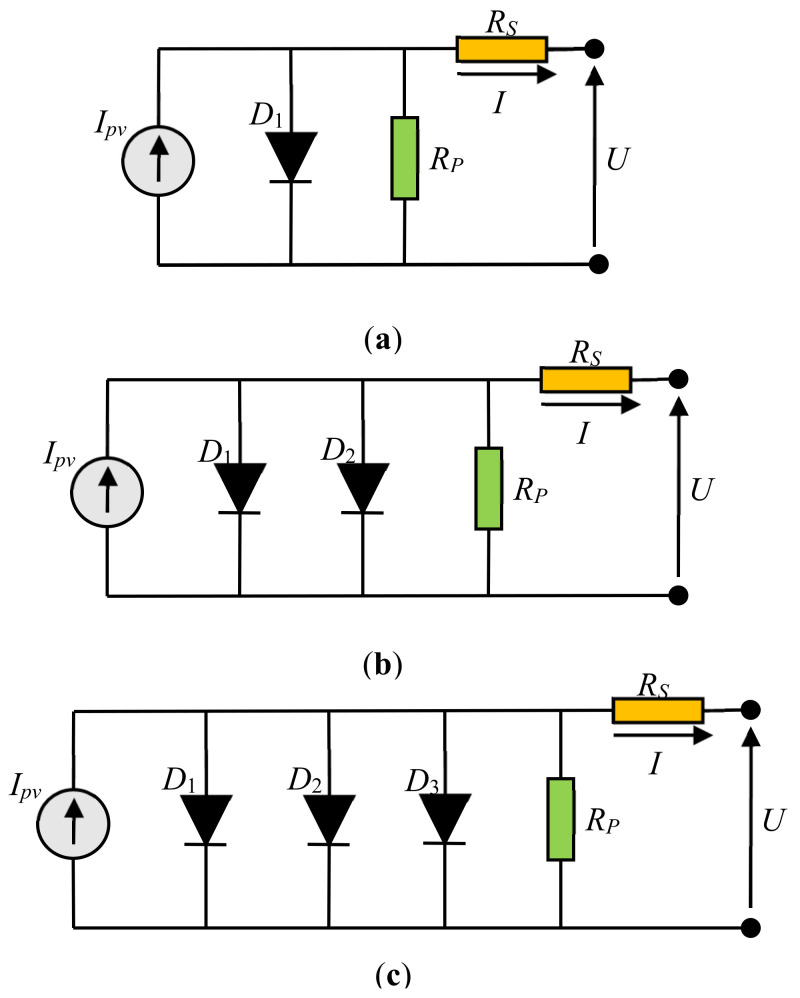
Common diode models of solar PV equivalent circuits: (**a**) SDM, (**b**) DDM, and (**c**) TDM.

Three-diode models of solar PV equivalent circuits can be found in the literature. The widely used and well-known solar cell model is the single-diode model (SDM), presented in Figure 1a. This model consists of four elements—an ideal current generator (*I_pv_*), diode (*D*), series resistance (*R_S_*), and parallel resistance (*R_P_*). Besides, the double-diode model (DDM) and triple-diode model (TDM), presented in Figure 1b,c, respectively, are widely used in the literature. Unlike SDM, these models consist of two (*D*_1_ and *D*_2_) and three diodes (*D*_1_, *D*_2_, and *D*_3_) [18,35,49,50,51,52].

The current (*I*)–voltage (*U*) relationship of these models can be described for SDM, DDM, and TDM as given in (1)–(3), respectively. In these equations, *I_pv_* denotes the photo-generated current. *I*_01_, *I*_02_, and *I*_03_ represent the reverse saturation current of the three diodes, respectively. *n*_1_, *n*_2_, and *n*_3_ represent the ideality factors of the diodes, respectively, and *V_th_* is the thermal voltage, which equals K_B_*T*/*q*, where K_B_ is the Boltzmann constant, *q* is the charge of the electron, and *T* is the temperature in Kelvin.

(1) $$I=I_{pv}-I_{01}\left(e^{\frac{U+IR_{S}}{n_{1}V_{th}}}-1\right)-\frac{U+IR_{S}}{R_{P}}$$

(2) $$I=I_{pv}-I_{01}\left(e^{\frac{V+IR_{S}}{n_{1}V_{th}}}-1\right)-I_{02}\left(e^{\frac{V+IR_{S}}{n_{2}V_{th}}}-1\right)-\frac{U+IR_{S}}{R_{P}}$$

(3) $$I=I_{pv}-I_{01}\left(e^{\frac{V+IR_{S}}{n_{1}V_{th}}}-1\right)-I_{02}\left(e^{\frac{V+IR_{S}}{n_{2}V_{th}}}-1\right)-I_{03}\left(e^{\frac{V+IR_{S}}{n_{3}V_{th}}}-1\right)-\frac{U+IR_{S}}{R_{P}}$$

It is apparent that *I*–*U* expressions of the three models are transcendental, i.e., highly nonlinear.

For SDM, the analytical solution of the current as a function of voltage is given as follows:

(4) $$I=\frac{R_{P}\left(I_{pv}+I_{0}\right)-U}{R_{S}+R_{P}}-\frac{n_{1}V_{th}}{R_{S}}W(\alpha_{S})$$

where

(5) $$\alpha_{S}=\frac{I_{0}R_{P}R_{S}}{n_{1}V_{th}\left(R_{S}+R_{P}\right)}\exp\left(\frac{R_{P}\left(R_{S}I_{pv}+R_{S}I_{0}+U\right)}{n_{1}V_{th}\left(R_{S}+R_{P}\right)}\right)$$

where W represents Lambert’s W function.

The *I*–*U* expressions of both DDM and TDM do not have exact analytical solutions. However, in [10], an original iterative procedure for solving these nonlinear equations was proposed and tested. The iterative-based solution of the current as a function of the voltage for DDM is formulated as follows [10]:

(6) $$I=\frac{\left(I_{pv}+I_{01}+I_{02}-\frac{U}{R_{P}}-\frac{\Psi \left(1+\frac{R_{s}}{R_{p}}\right)}{\frac{R_{s}}{n_{1}V_{th}}}\right)}{1+\frac{R_{s}}{R_{p}}}$$

where Ψ is the solution of the nonlinear equation so that

(7) $$\alpha_{D}+\beta_{D}\exp\left(\delta_{D}\Psi \right)=\Psi \exp\left(\Psi \right)$$

(8) $$\alpha_{D}=\frac{\frac{R_{S}}{n_{1}V_{th}}}{\left(1+\frac{R_{S}}{R_{P}}\right)}I_{01}\exp\left(\frac{U}{n_{1}V_{th}}\right)\cdot \exp\left(\frac{\frac{R_{S}}{n_{1}V_{th}}\left(I_{pv}+I_{01}+I_{02}-\frac{U}{R_{P}}\right)}{\left(1+\frac{R_{S}}{R_{P}}\right)}\right)$$

(9) $$\beta_{D}=\frac{\frac{R_{S}}{n_{1}V_{th}}}{\left(1+\frac{R_{S}}{R_{P}}\right)}I_{02}\exp\left(\frac{U}{n_{2}V_{th}}\right)\cdot \exp\left(\frac{R_{s}}{n_{2}V_{th}}\frac{\left(I_{pv}+I_{01}+I_{02}-\frac{U}{R_{P}}\right)}{\left(1+\frac{R_{S}}{R_{P}}\right)}\right)$$

(10) $$\delta_{D}=1-\frac{n_{1}}{n_{2}}$$

Additionally, the iterative-based solution of the current as a function of the voltage for TDM is formulated as follows [10]:

(11) $$I=\frac{\left(I_{pv}+I_{01}+I_{02}+I_{03}-\frac{U}{R_{P}}-\frac{Ζ\left(1+\frac{R_{S}}{R_{P}}\right)}{\frac{R_{S}}{n_{1}V_{t}}}\right)}{1+\frac{R_{S}}{R_{P}}}$$

where Z is the solution of the nonlinear equation.

(12) $$\alpha_{T}+\beta_{T}\exp\left(\delta_{T}Ζ\right)+\gamma_{T}\exp\left(\sigma_{T}Ζ\right)=\mathrm{Ζexp}\left(Ζ\right)$$

(13) $$\alpha_{T}=\frac{\frac{R_{S}}{n_{1}V_{th}}}{\left(1+\frac{R_{S}}{R_{P}}\right)}I_{01}\exp\left(\frac{U}{n_{1}V_{th}}\right)\cdot \exp\left(\frac{\frac{R_{S}}{n_{1}V_{th}}\left(I_{pv}+I_{01}+I_{02}+I_{03}-\frac{U}{R_{P}}\right)}{\left(1+\frac{R_{S}}{R_{p}}\right)}\right)$$

(14) $$\beta_{T}=\frac{\frac{R_{S}}{n_{1}V_{th}}}{\left(1+\frac{R_{S}}{R_{P}}\right)}I_{02}\exp\left(\frac{U}{n_{2}V_{t}}\right)\cdot \exp\left(\frac{R_{S}}{n_{2}V_{th}}\frac{\left(I_{pv}+I_{01}+I_{02}+I_{03}-\frac{U}{R_{P}}\right)}{\left(1+\frac{R_{S}}{R_{P}}\right)}\right)$$

(15) $$\gamma_{T}=\frac{\frac{R_{S}}{n_{1}V_{th}}}{\left(1+\frac{R_{S}}{R_{P}}\right)}I_{03}\exp\left(\frac{U}{n_{3}V_{th}}\right)\cdot \exp\left(\frac{R_{S}}{n_{3}V_{th}}\frac{\left(I_{pv}+I_{01}+I_{02}+I_{03}-\frac{U}{R_{P}}\right)}{\left(1+\frac{R_{s}}{R_{P}}\right)}\right)$$

(16) $$\delta_{T}=1-\frac{n_{1}}{n_{2}}$$

(17) $$\sigma_{T}=1-\frac{n_{1}}{n_{3}}$$

## 3. Analytical Formulation of a New Six-Parameter Solar Cell Model: Improved Single-Diode Model (ISDM)

**Figure 2:**
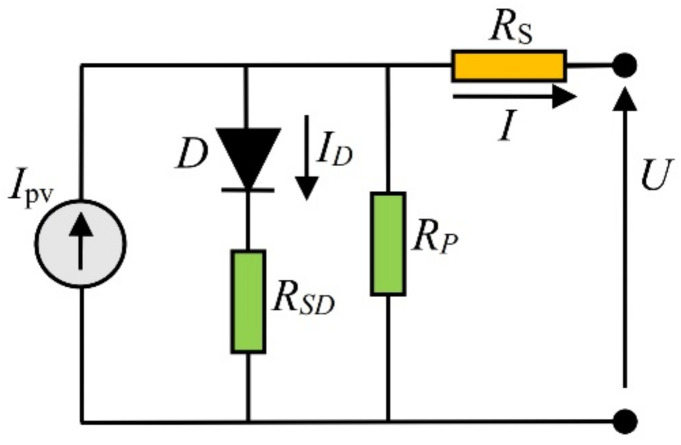
Improved solar PV equivalent circuit, ISDM.

A PV cell is a semiconductor device that converts sunlight into electricity [53]. However, light, i.e., the incoming photons to be absorbed, must have more incredible energy than the bandgap energy of the cell [54]. The absorbed photon generates pairs of mobile charge carriers (electron and hole), which are then separated by the structure of the device (*p*–*n* junction). This action produces a potential difference and thus creates an electrical current. Currently, semiconductor materials (usually silicon) in the *p*–*n* junction (diode) are commercially used to produce solar cells. The well-known Shockley equation gives the *I*–*U* characteristic of a *p*–*n* junction [54]. The current generated in the PV cell flows through a semiconductor material. However, different types of losses exist in a solar cell. In order to represent all series resistances, such as the resistance of the metal grid, contacts, and current-collecting wires, the single-diode morel consists of equivalent resistance *R_S_*, added in series with the ideal circuit model (parallel connection of ideal current generator and diode). On the other side, as the solar cells are made out of large-area wafers and from large thin-film material, second resistance, connected in parallel with the ideal device *R_P_*, also exists in the single-diode equivalent circuit. An improved SDM (ISDM) is proposed in this work to improve and collect all power energy losses in the solar cell. The proposed circuit of the ISDM is presented in Figure 2. Unlike the standard SDM, this model involves one additional resistance (*R_SD_*) connected in series with the diode to sufficiently express the power loss dissipation due to the current that flows through the *p*–*n* junction.

The equation that expresses the sum of currents in the ISDM is given as follows:

(18) $$I_{pv}=I_{D}+\frac{U+IR_{S}}{R_{P}}+I$$

where

(19) $$I_{D}=I_{0}\left(e^{\frac{V_{D}}{n_{1}V_{th}}}-1\right)$$

The voltage equation of this circuit is expressed as follows:

(20) $$V_{D}+R_{SD}I_{D}=U+IR_{S}$$

Hence, the expression of the current can be derived in the following form:

(21) $$I=\frac{R_{P}}{R_{S}+R_{P}}\left(I_{pv}+I_{01}-\frac{U}{R_{P}}-x\right)$$

where *x* is the solution of Lambert’s W function and is given in the following form:

(22) $$x=\beta \exp\left(-x\right)$$

where *β* is expressed as follows:

(23) $$\beta =I_{2}\left(\frac{b}{a}\right)\exp\left(\frac{b}{a}I_{1}\right)$$

so that

(24) $$\begin{array}{l} a=1+\frac{R_{S}}{R_{P}},\\ b=\frac{R_{S}}{nV_{t}}\left(1+\frac{R_{SD}}{R_{P}}+\frac{R_{SD}}{R_{S}}\right),\\ I_{1}=I_{pv}+I_{01}-\frac{U}{R_{P}},\\ I_{2}=I_{01}\exp\left(\frac{1}{nV_{t}}\left(U-R_{SD}I_{pv}+\frac{R_{SD}}{R_{P}}U\right)\right). \end{array}$$

**Figure 3:**
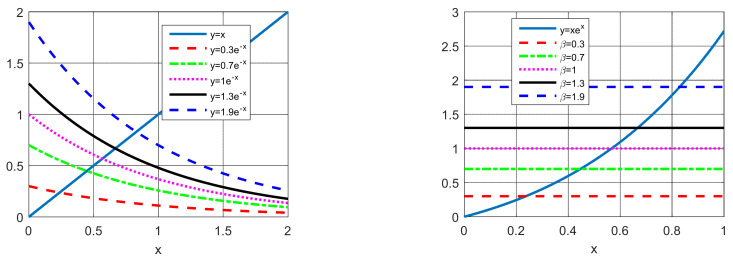
Lambert’s W function.

Given in Equation (22), Lambert’s W function is a nonlinear transcendental equation. This function is presented in Figure 3 for different values of *β*.

Different methods can solve this equation as it has become trendy in science. Many program packages (Matlab, Mathematica, Maple, and others) have implemented this equation. For instance, it can be solved using numerical techniques such as Frisch iteration, Newton–Raphson method, and others. Additionally, it can be solved analytically using the Taylor series or by using Special Trans Function Theory (STFT) [1,10,19,55,56].

Based on previous research [10,35] on the parameter estimation of PV equivalent circuits, it was clearly shown that the STFT has a significant advantage over the Taylor series. In this context, the analytical solution of the *I*–*U* relationship for the ISDM can be expressed as follows:

(25) $$I=\frac{R_{P}}{R_{S}+R_{P}}\left(I_{pv}+I_{01}-\frac{U}{R_{P}}-\beta \frac{\sum_{k=0}^{M}\frac{\beta^{k}{\left(M-k\right)}^{k}}{k!}}{\sum_{k=0}^{M+1}\frac{\beta^{k}{\left(M+1-k\right)}^{k}}{k!}}\right)$$

where *M* represents a positive integer. Additionally, the power–voltage relationship can be expressed as follows:

(26) $$P=U\cdot I=\frac{R_{P}U}{R_{S}+R_{P}}\left(I_{pv}+I_{01}-\frac{U}{R_{P}}-\beta \frac{\sum_{k=0}^{M}\frac{\beta^{k}{\left(M-k\right)}^{k}}{k!}}{\sum_{k=0}^{M+1}\frac{\beta^{k}{\left(M+1-k\right)}^{k}}{k!}}\right)$$

Therefore, the voltage corresponding to the maximum power delivered (*U_mp_*) by the cell/module can be determined as follows [57]:

(27) $${\left.\left(\frac{\partial P\left(U\right)}{\partial U}\right)\right|}_{U=U_{{}_{mp}}}=0$$

where

(28) $$P_{mp}=U_{mp}I_{mp}$$

Additionally, the current corresponding to the maximum power can be calculated easily, where *P_mp_* is the maximum power point of the solar cell/module.

## 4. Simulated Annealing (SA)–Manta Ray Foraging Optimization (MRFO)

The recently proposed Manta Ray Foraging Optimization (MRFO) is improved by the Simulated Annealing (SA) algorithm to formulate a novel hybrid algorithm called Simulated Annealing–Manta ray foraging optimization (SA-MRFO).

SA is usually used to hybridize standard metaheuristics algorithms [58,59]. It is a well-known and applicable algorithm. Due to its merits, it is implemented in Matlab and can be called by the function *simulannealbnd*. Algorithmically, SA is used when the search space is discrete. Additionally, its metaheuristic nature enables it to obtain approximate global or near-global solutions in an ample search space. SA has one main general characteristic: simulated annealing is preferable for problems where finding an approximate global optimum is more worthy than finding an accurate local optimum in a specific time. All the aspects mentioned above are the main reasons we developed the hybrid SA-MRFO algorithm in this paper. In the hybrid algorithm proposed in this paper (SA-MRFO), the SA algorithm is used to initialize the population of the MRFO.

Manta Ray Foraging Optimization (MRFO) is an algorithm realized by observing manta rays, the largest marine creatures [60,61]. This algorithm relies on three parts—chain, cyclone, and somersault foraging.

The first part of MRFO (chain foraging) focuses on the plankton position. This algorithm assumes that the best-found solution is plankton with a high concentration of manta rays. Specifically, the higher the plankton concentration, the better the position. At each iteration, each individual is updated with the best solution found to date and the solution in front of it. In a mathematical sense, the chain foraging model is represented as follows:

(29) $$x_{i}^{d}\left(t+1\right)=\left\{\begin{array}{l} x_{i}^{d}\left(t\right)+r\left(x_{best}^{d}\left(t\right)-x_{i}^{d}\left(t\right)\right)+\gamma \left(x_{best}^{d}\left(t\right)-x_{i}^{d}\left(t\right)\right),\;\;\;i=1\\ x_{i}^{d}\left(t\right)+r\left(x_{i-1}^{d}\left(t\right)-x_{i}^{d}\left(t\right)\right)+\gamma \left(x_{best}^{d}\left(t\right)-x_{i}^{d}\left(t\right)\right),i=2,\ldots ,N \end{array}\right.$$

where $x_{i}^{d}\left(t\right)$ is the position of the *i*th individual at time *t*, *r* and *r*_1_ are random numbers within the range of [0,1], while $x_{best}^{d}\left(t\right)$ denotes the plankton with a high concentration (best position). The chain foraging coefficient is denoted γ, which is expressed as $\gamma =2r\sqrt{\left|\log\left(r\right)\right|}$.

The second part of MRFO (cyclone foraging) is oriented on a school of manta rays. Namely, when a school of manta rays recognizes a patch of plankton, they will form a long foraging chain. Furthermore, they will swim toward the food in a spiral movement. The mathematical equation that expresses the spiral action of manta rays is the same as the expression given in (29), except that the cyclone foraging coefficient (γ) is expressed as γ=2er1T−t+1Tsin2πr1, where *T* denotes the maximum number of iterations. The reference position is the food, where all individuals orient towards it. Iteratively, each individual looks for a better position around it. In this sense, each individual has an opportunity to find itself in a random position. Mathematically, a change in the position is expressed as follows:

(30) $$x_{i}^{d}\left(t+1\right)=\left\{\begin{array}{l} x_{rand}^{d}+r\left(x_{rand}^{d}-x_{i}^{d}\left(t\right)\right)+\beta \left(x_{rand}^{d}-x_{i}^{d}\left(t\right)\right),\;\;\;i=1\\ x_{rand}^{d}+r\left(x_{i-1}^{d}\left(t\right)-x_{i}^{d}\left(t\right)\right)+\beta \left(x_{rand}^{d}-x_{i}^{d}\left(t\right)\right),i=2,\ldots ,N \end{array}\right.$$

where $x_{rand}^{d}$ is a randomly produced position in the search space. $Lb^{d}$ and $Ub^{d}$ denote the lower and upper boundaries of the decision variables.

**Figure 4:**
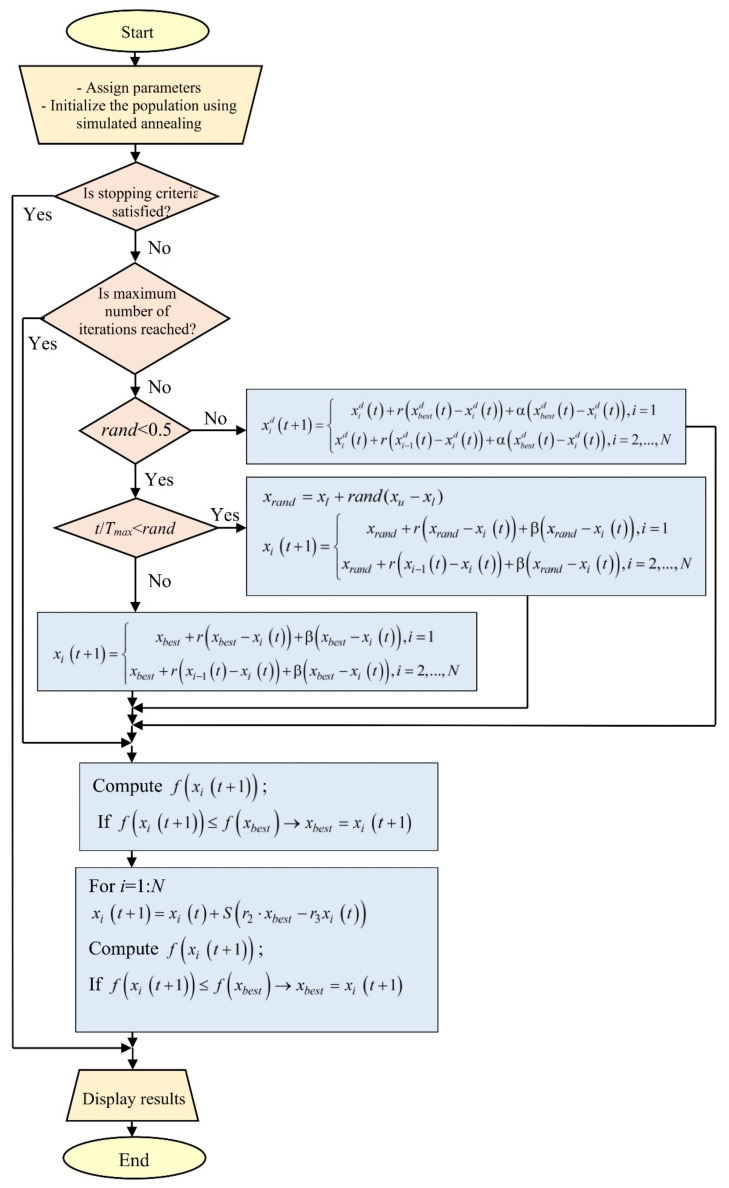
Flowchart of the proposed algorithm.

The third part of MRFO defines the movement of each individual in a new search domain located between the current position and its symmetrical position around the best position found to date (somersault foraging), in which the position of the food is viewed as a pivot. Each individual tends to swim around the pivot to reach a new position. Thus, each individual updates its position around the best position found. The mathematical model of this part can be expressed as follows:

(31) $$x_{i}^{d}\left(t+1\right)=x_{i}^{d}\left(t\right)+2\left(r_{2}\cdot x_{best}^{d}-r_{3}x_{i}^{d}\left(t\right)\right)$$

where *r*_2_ and *r*_3_ are random numbers in [0, 1]. The flowchart of the SA-MRFO algorithm is presented in Figure 4.

## 5. Results and Discussion

The results obtained using the proposed algorithm to estimate the intrinsic parameters of the addressed equivalent circuit models are presented in this section.

For parameter estimation, the minimization of the expression given in (32) that represents the root-mean-square error (*RMSE*) between the solar PV cell’s measured and calculated output current was used.

(32) $$RMSE=\sqrt{\frac{1}{N_{p}}\sum_{i=1}^{N_{p}}{\left(I_{i}^{meas}-I_{i}^{calc}\right)}^{2}}$$

The goal of the estimation process was to find the appropriate value of the solar cell parameters to minimize the *RMSE* between simulated and measured solar cell current values. In this equation, *N_p_* represents the number of the measured points, while $I_{i}^{meas}$ and $I_{i}^{calc}$ represent the measured and estimated solar cell current at point *i*, respectively.

The software tool used to estimate the intrinsic parameters of the PV cells was MATLAB 2018a. The computing tasks were implemented on a laptop PC with Intel(R) Core (TM) i3-7020U CPU @2.30 GHz and 4 GB RAM.

### 5.1. RTC France Solar Cell

A well-known commercial silicon solar cell called RTC. France is used to validate the effectiveness of the proposed algorithm and the accuracy of the ISDM. The RTC France solar cell is a benchmark cell usually used in testing the performance of optimization algorithms, with 26 pairs of current–voltage points available under test conditions of 1000 W/m^2^ irradiance and 33 °C temperature. This is why this solar cell is suitable for a fair comparison with all other algorithms, i.e., the results presented in the literature.

The results obtained for the ISDM of the RTC France cell using the proposed algorithm under the mentioned test conditions are shown in Table 1. Besides, the results presented for the SDM under the same test conditions are presented in the same table.

Table 2 shows the literature results (parameters and *RMSE* values) obtained for the RTC France solar cell (SDM, DDM, and TDM). It should be noted that *RMSE* values that are not presented in the methods addressed in Table 2 were calculated using Equation (32). Table A1 in Appendix A shows the parameters of the solar RTC France cell using the methods presented in Table 2. Acronyms of the algorithms presented in Table 2 are given in the list of abbreviations.

**Figure 5:**
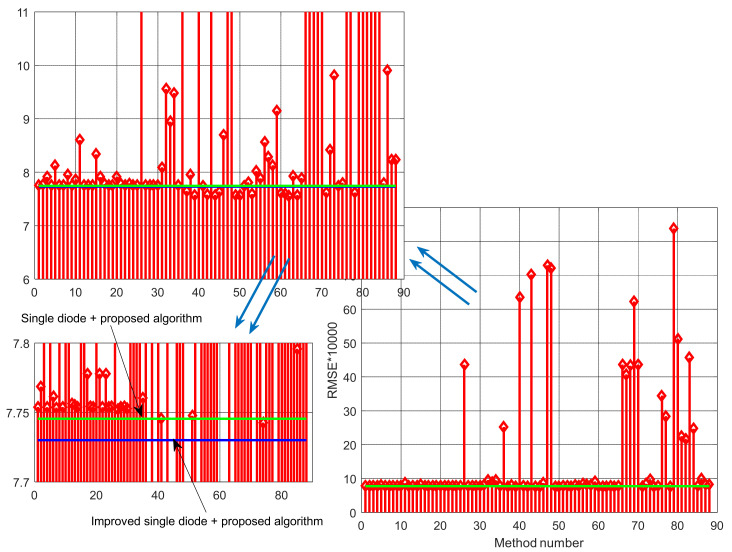
Visualization of the calculated *RMSE* values using the different methods presented in Table 2.

A few conclusions can be reached by observing the results presented in Table 1 and Table 2. First, the proposed algorithm is superior to many other compared algorithms in terms of the calculated *RMSE*. Second, the effectiveness of the proposed solar cell model, ISDM, is apparent as the calculated *RMSE* value is lower than all algorithms used in the literature for the parameter estimation of the SDM of the RTC France cell. Third, the proposed model and algorithm enable parameter estimation, giving lower *RMSE* values than many of the results reported in the literature, even for DDM and TDM of the RTC France cell. The visualization of the calculated *RMSE* values using the different methods presented in Table 2 is depicted in Figure 5. It indicates that the proposed method and circuit model enable obtaining better results than other models and algorithms.

**Figure 6:**
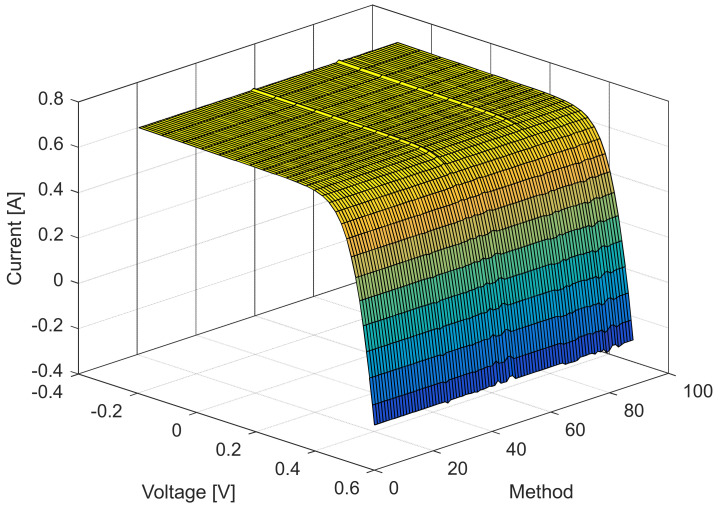
Current–voltage characteristics for the methods listed in Table 2.

**Figure 7:**
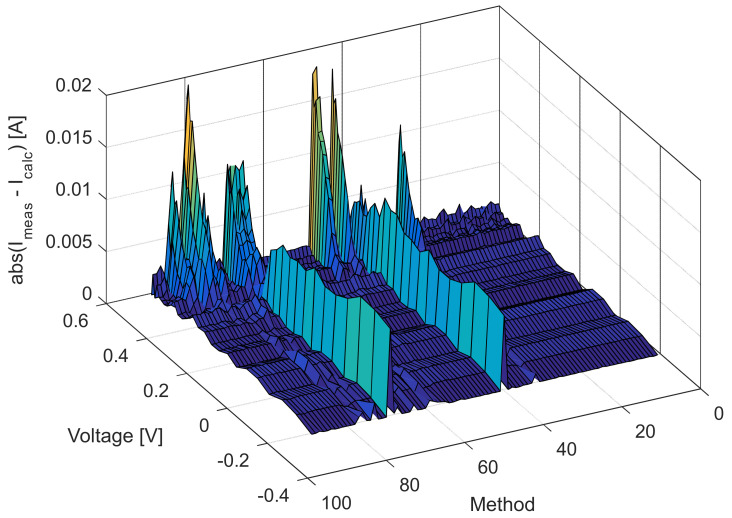
Difference between the measured and calculated current values for the methods listed in Table 2.

**Figure 8:**
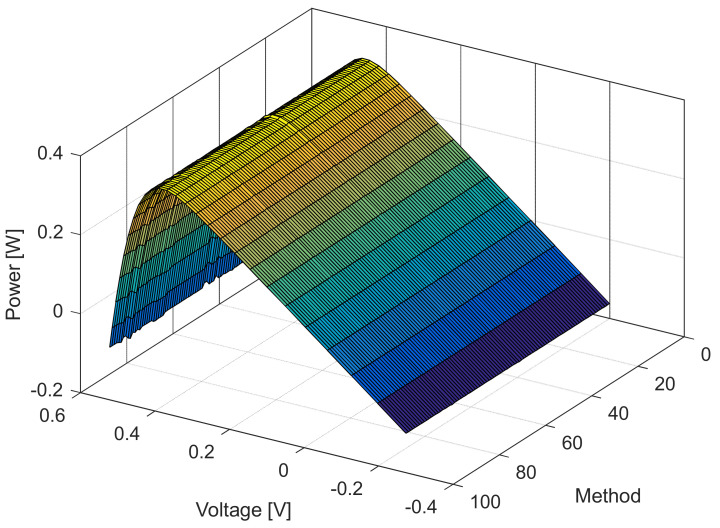
Power–voltage characteristics for the methods listed in Table 2.

**Figure 9:**
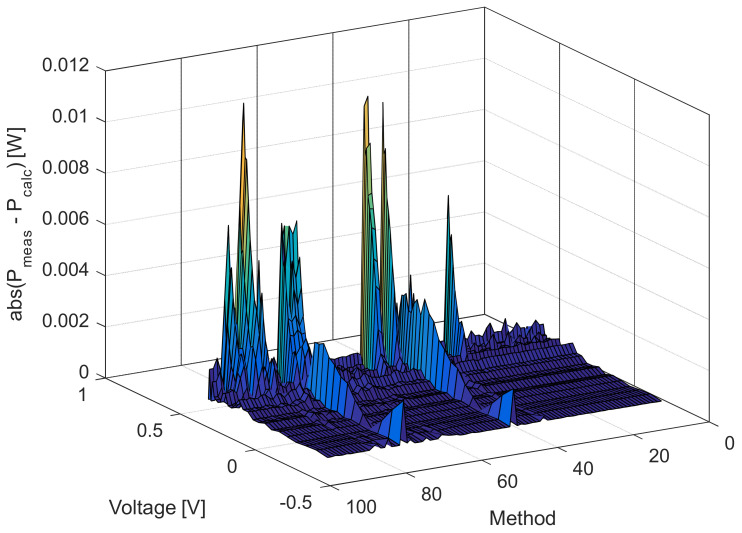
Difference between the measured and calculated power values for the methods listed in Table 2.

Figure 6, Figure 7, Figure 8 and Figure 9 illustrate current/power versus voltage characteristics and their corresponding errors. From the presented graphs, it is clear, at first glance, that there are no differences between the explored curves for all methods given in the available literature. However, observing the three-dimensional graphs of the error for both current and power, it can be seen that some methods give a minimal error value for all voltage values, while the error in other methods is high. The error, i.e., the difference between the measured and calculated value of current (or power), is specifically noticeable for large voltage values (close to the no-load voltage). The current error is almost negligible for low voltage values in all models.

**Figure 10:**
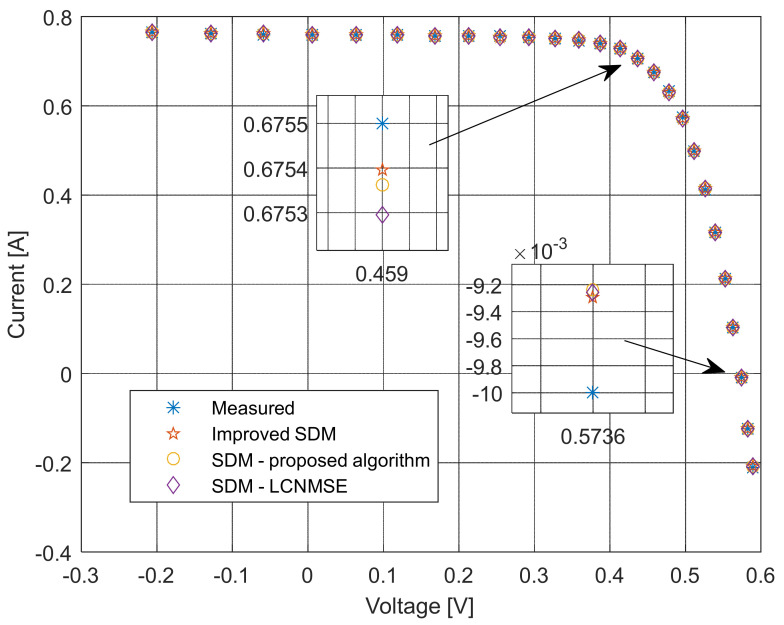
Current–voltage characteristics using different methods for both SDM and ISDM.

**Figure 11:**
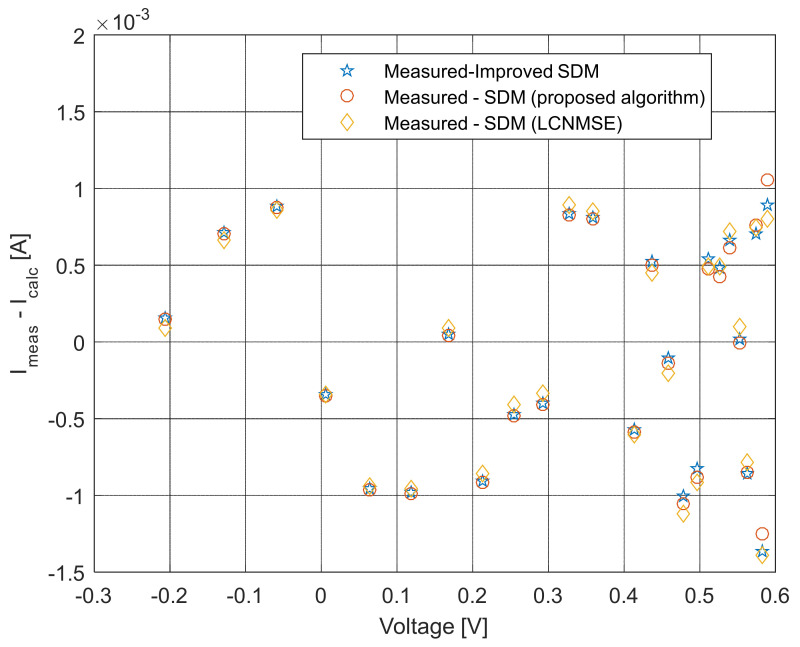
Difference between the measured and calculated current values using different methods for both SDM and ISDM.

**Figure 12:**
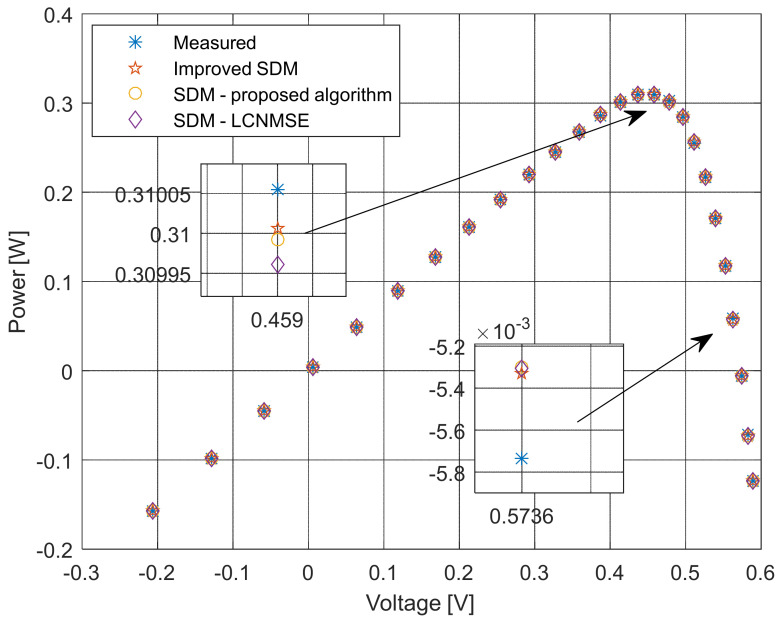
Power–voltage characteristics using different methods for both SDM and ISDM.

**Figure 13:**
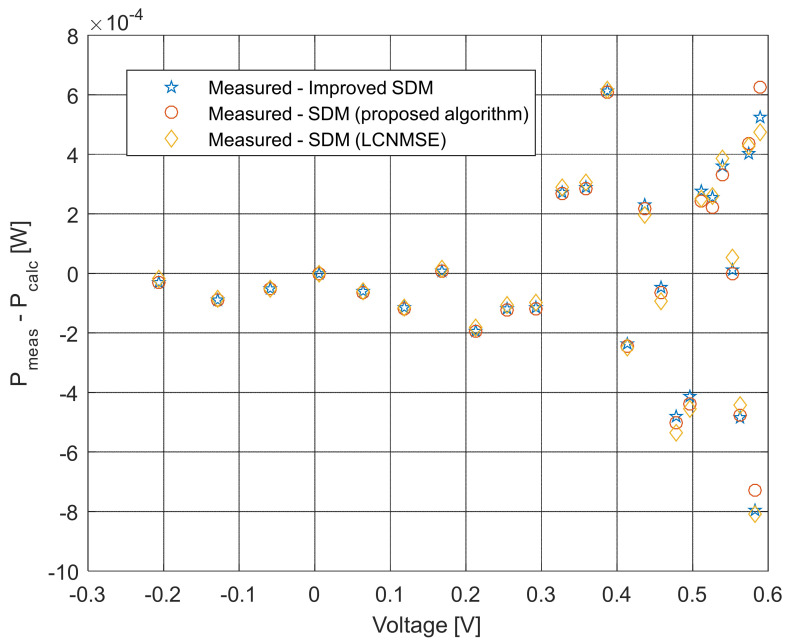
Difference between the measured and calculated power values using different methods for both SDM and ISDM.

The current–voltage and power–voltage characteristics and corresponding errors value for the proposed ISDM and the standard SDM, whose parameters were determined by the proposed algorithm and Laplacian Nelder–Mead spherical evolution (LCNMSE) [18], are illustrated in Figure 10, Figure 11, Figure 12 and Figure 13. It is evident that the results match well. Moreover, for a few particularly zoomed points, it is clear that the proposed model provides the possibility of better fitting the measured and simulated curve.

To confirm the accuracy and applicability of the proposed model of solar cells, we also compared the *RMSE* values obtained by applying the proposed model and algorithm with the results obtained using the deterministic methods described in [63] for the RTC France solar cell. Four different methods were used for the comparison—Laudani et al.’s solution [64], Cardenas et al.’s solution [65], Two-Step Linear Least-Squares (TSLLS) method [66], and TSLLS with refinement [66].

**Figure 14:**
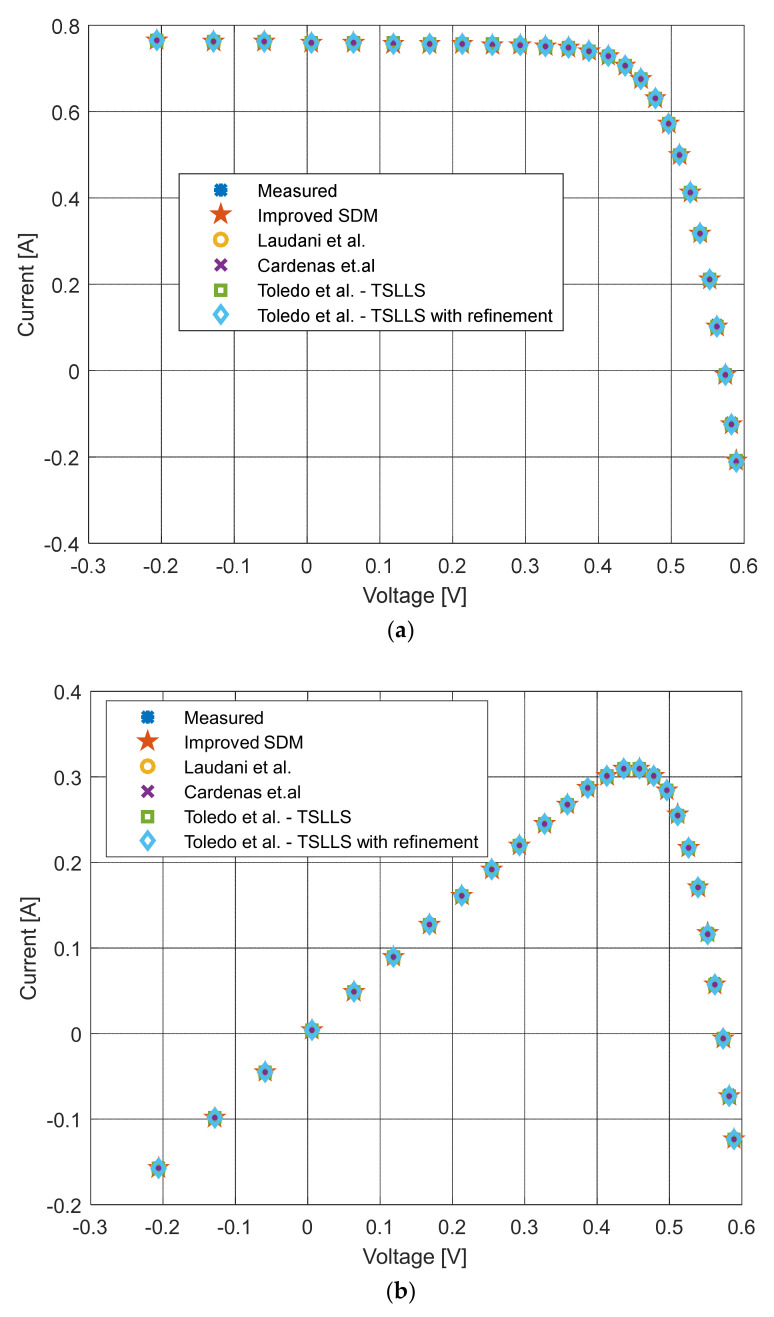

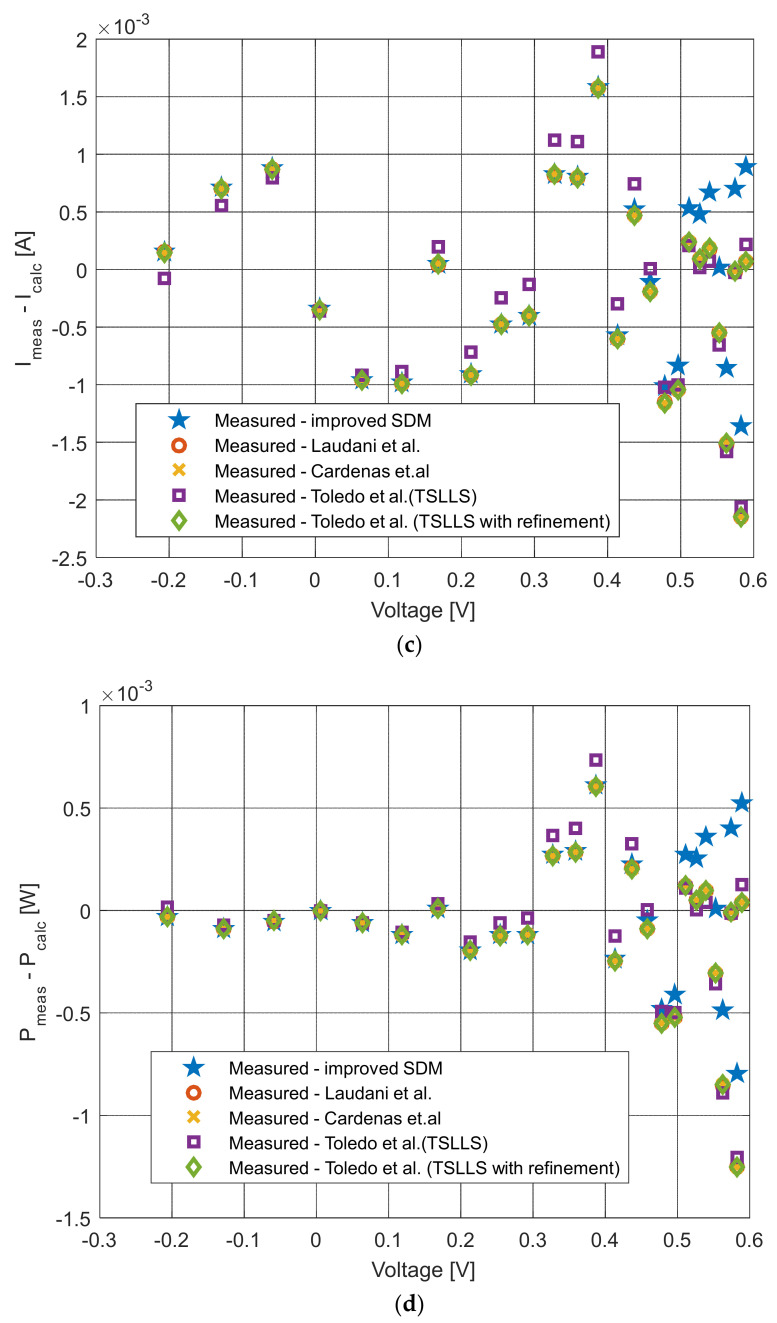
Comparison with the deterministic methods described in [63,64,65,66] for the RTC France solar cell using different methods for both SDM and ISDM: (**a**) current–voltage characteristics, (**b**) power–voltage characteristics, (**c**) difference between the measured and calculated current values, and (**d**) difference between the measured and calculated power values.

The current–voltage characteristics, power–voltage characteristics, difference between the measured and calculated current values, and difference between the measured and calculated power values using different methods for both SDM and ISDM are shown in Figure 14.

The obtained results are shown in Table 3, in which the *RMSE* values taken from [63] and the calculated *RMSE* values are presented. The minor difference between the values is due to the difference in the value of the thermal voltage, for which this work uses the values of the Boltzmann constant and elementary charge defined in the International System of Units (SI). Using the proposed method for calculating *RMSE* and considering the same thermal voltage value given in [63], we obtained the same *RMSE* values.

### 5.2. Solarex MSX 60 Solar Module

**Figure 15:**
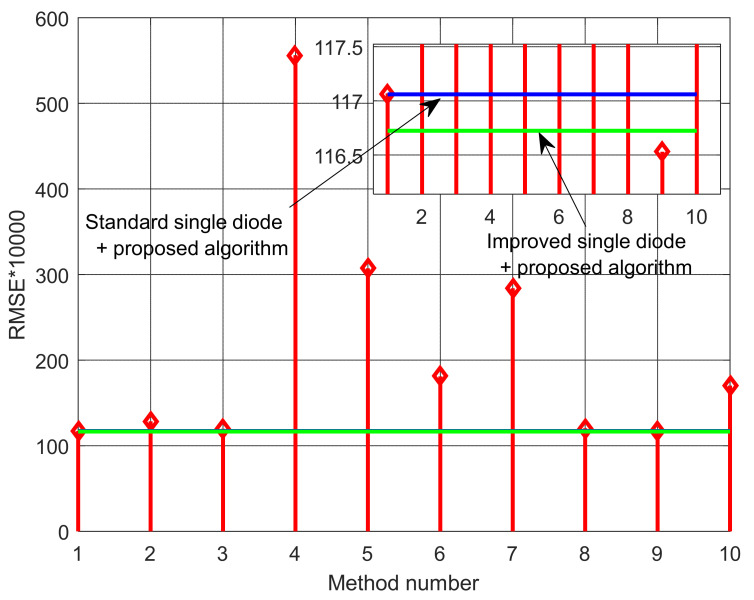
Visualization of the calculated *RMSE* values using the different methods presented in Table 5.

A similar investigation for the well-known Solarex MSX 60 module was also conducted. Namely, the parameters of the SDM and ISDM were determined by applying the proposed algorithm. The obtained results are presented in Table 4, and the difference in the obtained *RMSE* values is visualized in Figure 15. An overview of the known results in the literature for the MSX 60 solar module, described via the equivalent SDM, DDM, and TDM circuits, is shown in Table 5. Table A2 in Appendix A shows the Solarex MSX 60 module parameters using the methods presented in Table 5. From these results, it can be concluded that the proposed model is accurate, and the proposed algorithm is highly efficient for estimating the parameters of solar modules.

**Figure 16:**
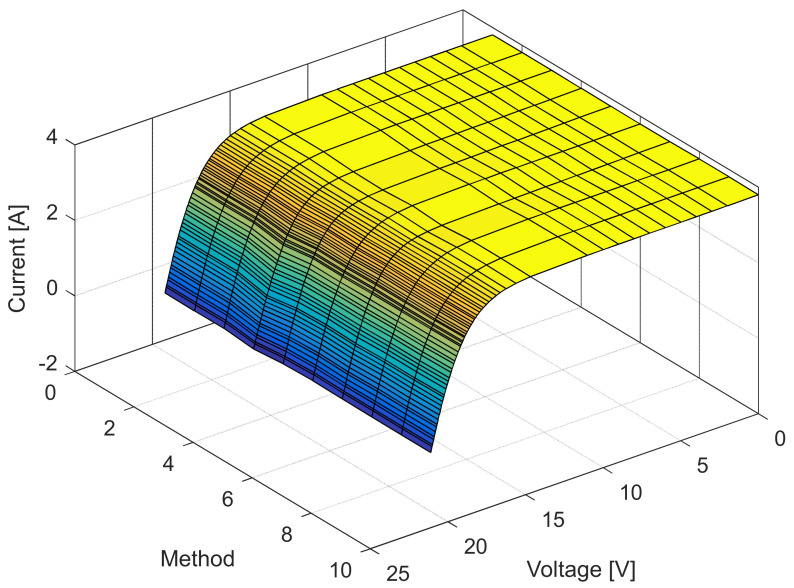
Current–voltage characteristics for the methods listed in Table 5.

**Figure 17:**
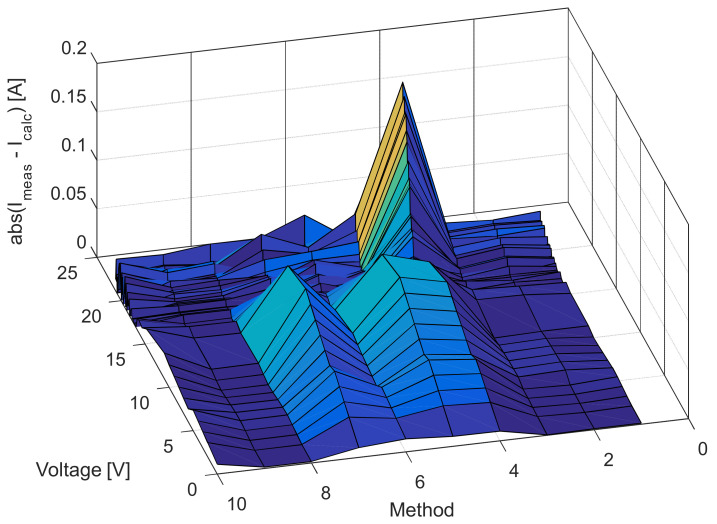
Difference between the measured and calculated current values for the methods listed in Table 5.

**Figure 18:**
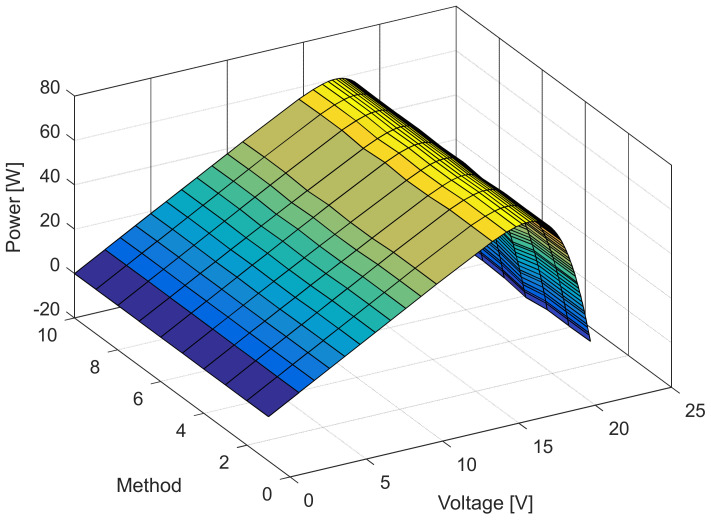
Power–voltage characteristics for the methods listed in Table 5.

**Figure 19:**
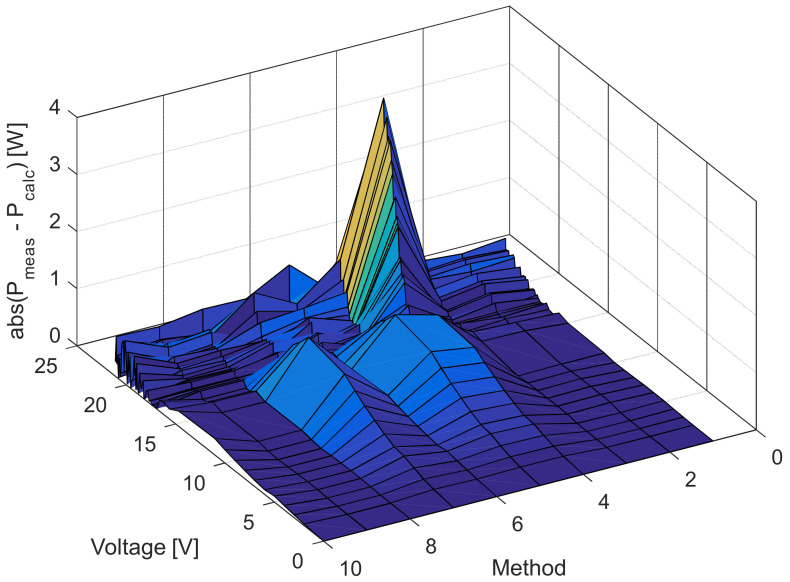
Difference between the measured and calculated power values for the methods listed in Table 5.

**Figure 20:**
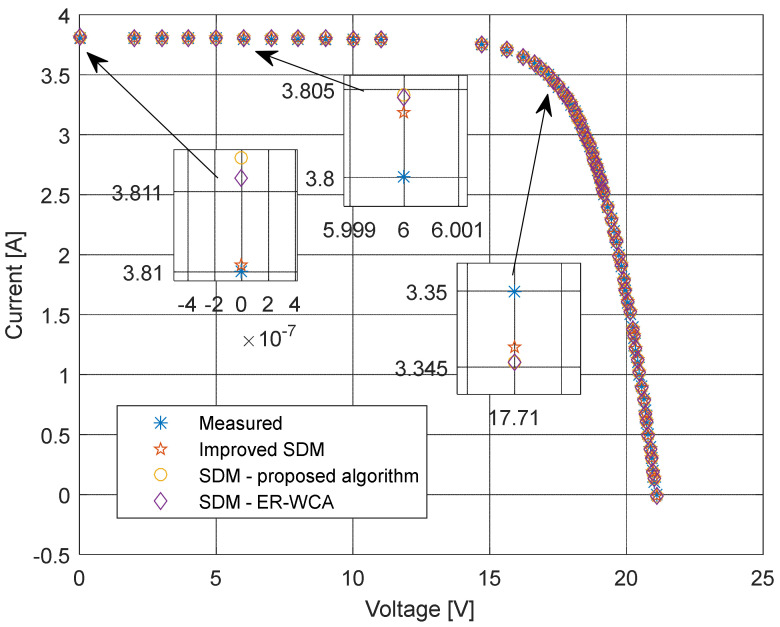
Current–voltage characteristics obtained using different methods for both SDM and ISDM.

**Figure 21:**
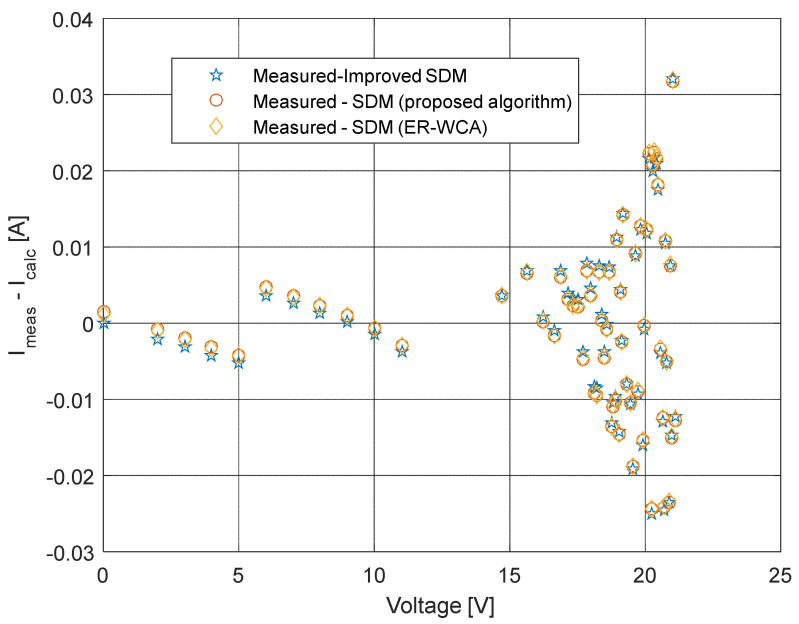
Difference between the measured and calculated current values using different methods for both SDM and ISDM.

**Figure 22:**
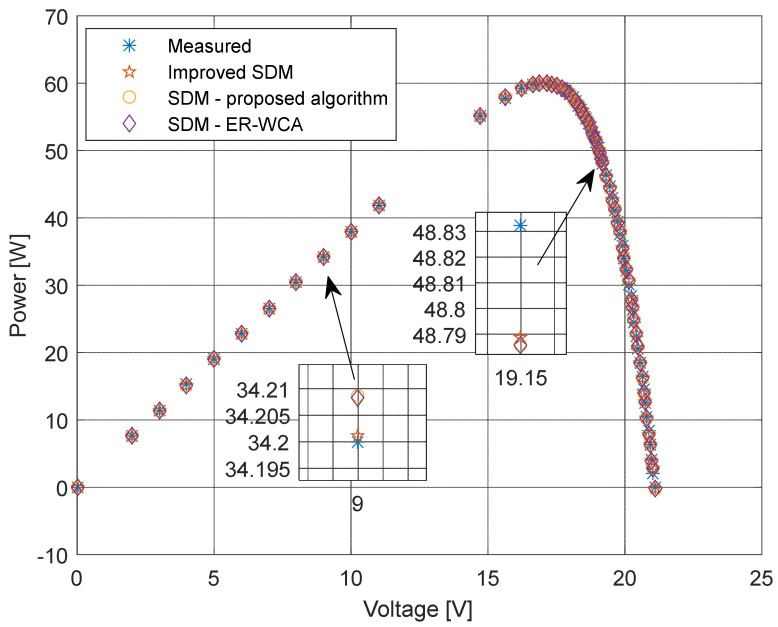
Power–voltage characteristics obtained using different methods for both SDM and ISDM.

**Figure 23:**
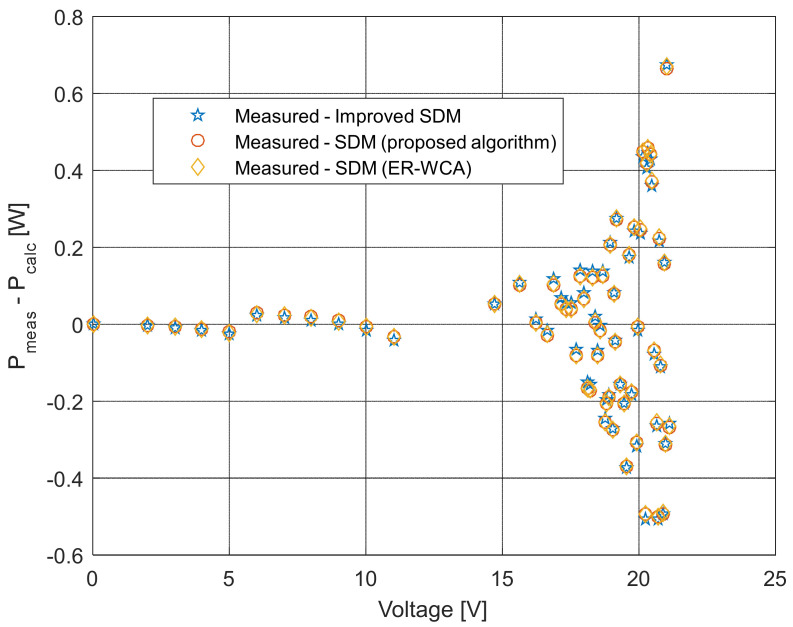
Difference between the measured and calculated power values using different methods for both SDM and ISDM.

The current and power change for different voltage values obtained using the methods considered are shown in Figure 16, Figure 17, Figure 18 and Figure 19. Based on the results obtained, it is clear that there are some differences between the measured and calculated values of current and power, especially for high voltage values. The current–voltage and power–voltage characteristics for the proposed model of solar cells and the standard single diode model, whose parameters were determined by the proposed algorithm and evaporation rate-based water cycle algorithm (ER-WCA), are depicted in Figure 20, Figure 21, Figure 22 and Figure 23. From the presented results, it is clear that the measures superbly match and that the proposed circuit, without doubt, increases the modeling accuracy of the solar cells.

### 5.3. Effectiveness of the Algorithm

**Figure 24:**
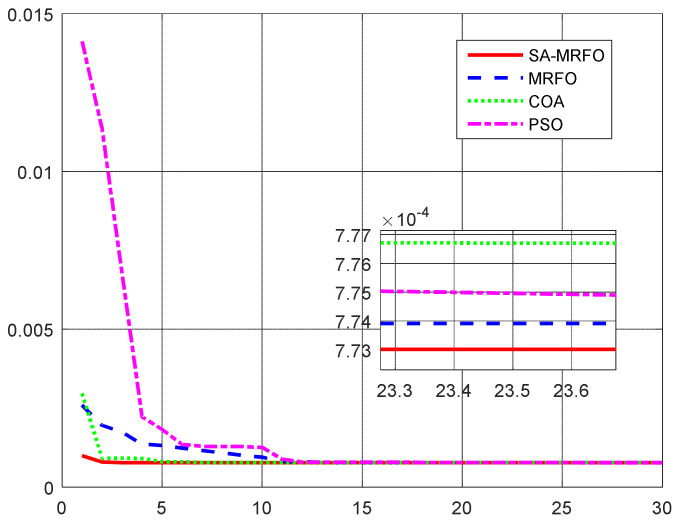
Comparison of the convergence curves for different optimization algorithms.

To further analyze the performance of the proposed algorithm, a comparison of the convergence characteristics of the proposed algorithm and some of known algorithms in the literature was performed. Additionally, the statistical measures of the presented algorithm results were performed and reported in Table 6 and Table 7. Additionally, Figure 24 shows the convergence rates of the different algorithms toward the optimal solution [67].

Based on all the presented results, it is evident that the proposed model of solar cells improves the accuracy of fitting current–voltage characteristics without increasing the computational complexity of the calculation. On the other side, the proposed algorithm estimates parameters with greater accuracy than many previously known methods.

From Figure 24, it is clear that the proposed hybrid algorithm contributes to better convergence towards the optimal solution. Additionally, statistical tests show that mean, median, and standard deviations have better features than the other considered algorithms. Based on the above, it is clear that the proposed algorithms have exceptional statistical features compared to different algorithms.

## 6. Experimental Application

**Figure 25:**
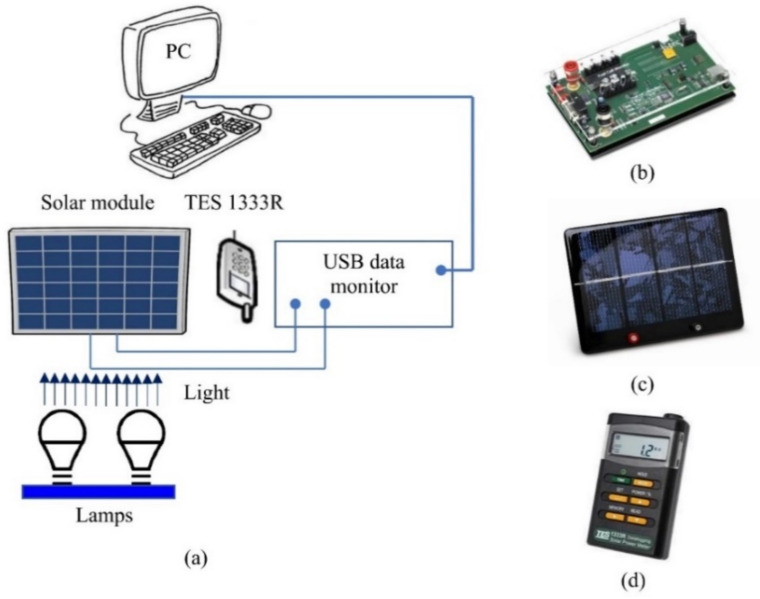
Experimental setup: (**a**) connection diagram, (**b**) USB data monitor, (**c**) solar module, and (**d**) device for insolation measurement.

The measurement of current–voltage characteristics of a solar laboratory module manufactured by Clean Energy Trainer was undertaken to validate the performance of the proposed model experimentally. The experimental setup—a connection diagram of the measuring equipment that includes a personal computer (PC), solar module, an insolation source lamp, an insolation measuring device (TES 1333R) with a resolution 0.1 W/m^2^, and a USB data monitor for data acquisition and processing of all components—is depicted in Figure 25. The measurements were taken in October 2021.

**Figure 26:**
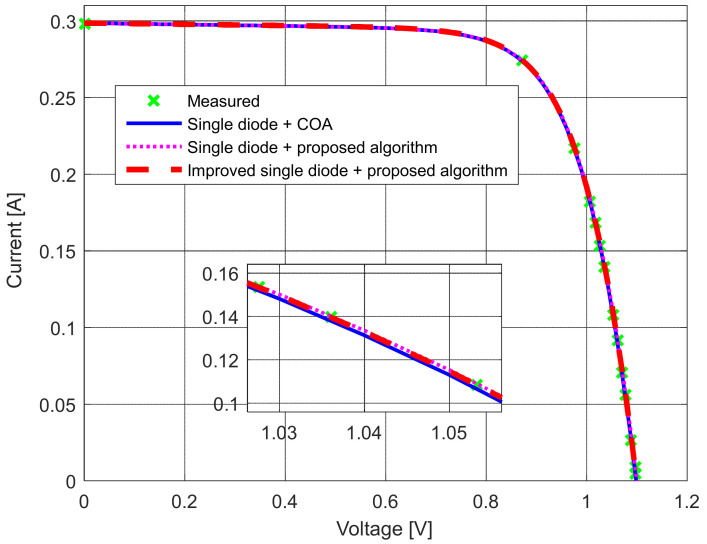
The measured and simulated *I–U* characteristics for the standard and modified single-diode models.

**Figure 27:**
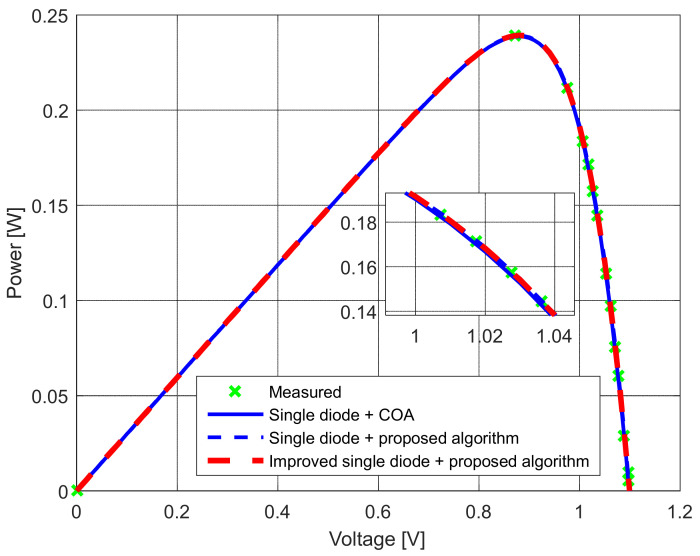
The measured and simulated *P–U* characteristics for the standard and modified single-diode models.

The measurements were performed with numerous replicates and careful monitoring of the module’s temperature. The solar module temperature was kept unchanged in all experiments, around 39 °C. First, the *I*–*U* and *P*–*U* characteristics were measured at 1300 W/m^2^ (as depicted in Figure 26 and Figure 27). Afterward, the parameters of the solar PV equivalent circuits were estimated using the proposed algorithm for the standard and modified single-diode models. The obtained results are shown in Table 8. They are also compared with the results presented in [10] that were determined using the COA algorithm.

**Figure 28:**
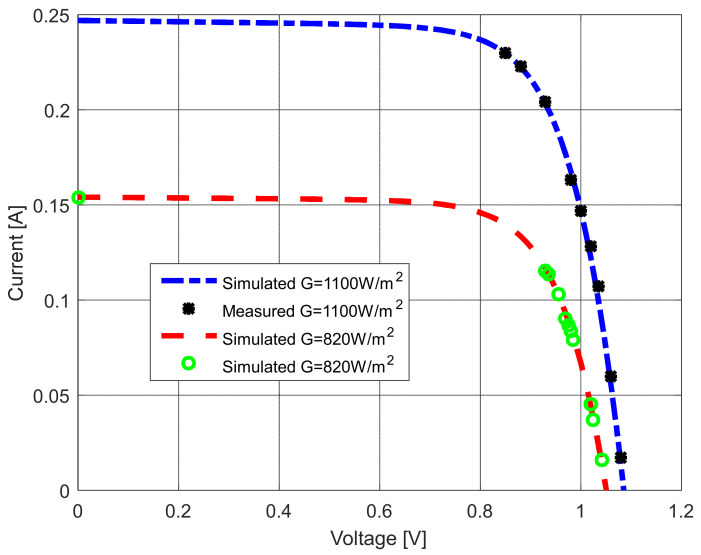
The measured and simulated *I–U* characteristics using different irradiance values.

**Figure 29:**
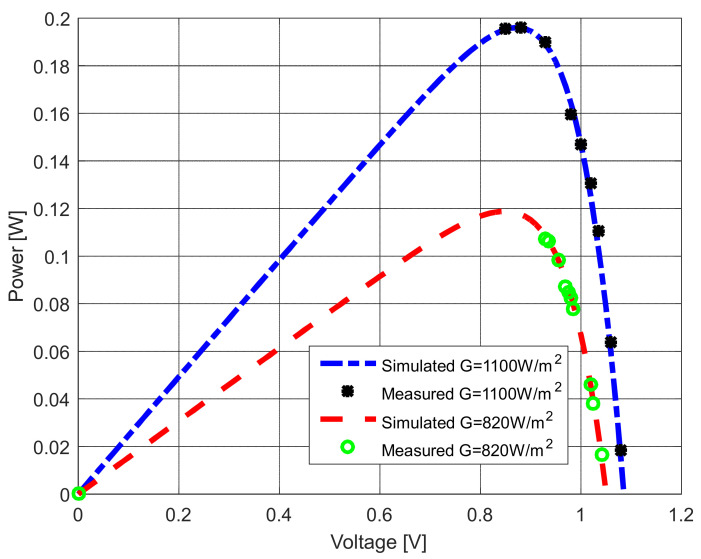
The measured and simulated *P–U* characteristics using different irradiance values.

Further, two additional current–voltage characteristics were recorded to check the accuracy of the results. This measurement was taken for two irradiance (*G*) values—1100 W/m^2^ and 830 W/m^2^. The measured and estimated current–voltage characteristics of the solar modules are compared in Figure 28 and Figure 29.

It is apparent that the results are close to each other. Additionally, the agreement between both measured and estimated characteristics is remarkable for all investigated cases. Note that the change of parameters with the different irradiance values was taken from [68] for calculation purposes.

## 7. Conclusions

This paper proposed an amended single-diode model of equivalent circuit models of solar cells. This amendment was realized by adding resistance in series with the diode of the single-diode model to represent power loss dissipation better. The mathematical expression of the current–voltage characteristic of the proposed model was derived. An analytical solution of the transcendental expression was developed in terms of Lambert’s W function and was further solved using the STFT. In addition, a novel hybrid algorithm for solar cell parameters estimation was proposed for the parameter estimation of the standard and improved single-diode models.

The proposed solar cell model enables the better fitting of the measured current–voltage characteristics. This statement was proved by comparing the estimated characteristics with many characteristics obtained for the parameters of solar cells available in the literature. Moreover, the proposed solar cell model and algorithm were tested on two well-known solar cells/modules. The experimental measurement of the current–voltage characteristics of a solar laboratory module was also realized. The results undoubtedly show that the proposed model and algorithm provide better accuracy and efficiency than traditional models.

Moreover, the accuracy obtained by applying the proposed model is even better than many of the accuracy values obtained by using more complex models—DDM and TDM. Finally, this research aimed to develop a good base for the further investigation of new generations of solar cell models and the implementation of efficient optimization algorithms to solve the parameter estimation problem.

In future work, considerable attention will be paid to developing accurate two-diode and three-diode models of solar cells using additional resistors as an amendment to the single-diode model proposed in this work.

## Figures and Tables

**Table 1:** Lower and upper bounds of the parameters and the results obtained for the RTC France solar cell for both SDM and ISDM.

Parameter	Bounds		Model	
	Lower	Upper	SDM	ISDM
*I_pv_* (A)	0.4	0.8	0.760787000	0.76079199200
*I*_01_ (μA)	0.1	0.5	0.310684200	0.31215026600
*n*_1_	1.0	1.7	1.477281000	1.47744988425
*R_S_* (Ω)	0.01	0.05	0.036581000	0.03638798313
*R_P_* (Ω)	40	70	52.87890000	52.8792013970
*R_SD_* (mΩ)	0.001	0.3	-	0.15054000000
*RMSE*			7.74549919 × 10^−4^	7.73001979 × 10^−4^

**Table 2:** *RMSE* calculation for the solar RTC France cell using various algorithms.

Method	Ref.	Algorithm	Model	*RMSE*	Method	Ref.	Algorithm	Model	*RMSE*
1	Proposed	SA-MRFO	ISDM	0.000773002	46	[40]	EOTLBO	DDM	0.000757585
2	Proposed	SA-MRFO	SDM	0.000774549	47	[26]	EHHO	DDM	0.000764087
3	[18]	LCNMSE	SDM	0.000775390	48	[32]	CSO	DDM	0.000869770
4	[20]	EO	SDM	0.000776865	49	[33]	R-II	DDM	0.007293813
5	[21]	WHHO	SDM	0.000791502	50		R-III	DDM	0.007220113
6	[4]	CNMSMA	SDM	0.000775388	51	[42]	COA	DDM	0.000757686
7	[24]	GAMNU	SDM	0.000812621	52	[12]	PGJAYA	DDM	0.000756809
8	[39]	GSK	SDM	0.000776134	53		GOTLBO	DDM	0.000774762
9	[7]	EABOA	SDM	0.000775354	54		JAYA	DDM	0.000781225
10	[5]	SMA	SDM	0.000795243	55		STLBO	DDM	0.000759274
11	[40]	EOTLBO	SDM	0.000775391	56		TLABC	DDM	0.000802419
12	[26]	EHHO	SDM	0.000786704	57		CLPSO	DDM	0.000788578
13	[32]	CSO	SDM	0.000860194	58		BLPSO	DDM	0.000856308
14	[33]	R-II	SDM	0.000775645	59		DE/BBO	DDM	0.000828349
15		R-III	SDM	0.000775557	60	[47]	CLPSO	DDM	0.000813110
16	[27]	CPMPSO	SDM	0.000775393	61		BLPSO	DDM	0.000915079
17	[46]	HCLPSO	SDM	0.000833742	62		IJAYA	DDM	0.000761222
18	[28]	FPSO	SDM	0.000791115	63		SFS	DDM	0.000759762
19	[6]	ITLBO	SDM	0.000777792	64		pSFS	DDM	0.000755741
20	[47]	pSFS	SDM	0.000775415	65	[30]	FA	DDM	0.000793077
21	[43]	ISCA	SDM	0.000775389	66		HFAPS	DDM	0.000757633
22	[41]	ILCOA	SDM	0.000791666	67		ABC	DDM	0.000789395
23	[22]	MADE	SDM	0.000777792	58	[25]	ELPSO	DDM	0.004363207
24	[42]	COA	SDM	0.000775383	69		BSA	DDM	0.004074336
25	[12]	PGJAYA	SDM	0.000777792	70		ABC	DDM	0.004332926
26	[11]	GAMS	SDM	0.000775395	71		GA	DDM	0.006223578
27	[44]	BHCS	SDM	0.000775415	72		ELPSO	DDM	0.004363207
28	[29]	MPSO	SDM	0.004359909	73	[48]	SATLBO	DDM	0.000762457
29	[30]	HFAPS	SDM	0.000775248	74	[62]	CWOA	DDM	0.000842359
30	[23]	ISCE	SDM	0.000775391	75	[9]	IJAYA	DDM	0.000980735
31	[45]	TLABC	SDM	0.000775416	76		LETLBO	DDM	0.000774275
32	[34]	ER-WCA	SDM	0.000775291	77		LBSA	DDM	0.000780352
33	[31]	MSSO	SDM	0.000809159	78	[8]	BPFPA	DDM	0.003447871
34	[8]	BPFPA	SDM	0.000955513	79	[21]	WHHO	TDM	0.002839541
35	[36]	WDO	SDM	0.000894818	80	[4]	CNMSMA	TDM	0.000762096
36	[41]	CWOA	SDM	0.000948338	81	[5]	SMA	TDM	0.008381759
37	[9]	IJAYA	SDM	0.000776055	82	[33]	R-II	TDM	0.005125476
38	[7]	EABOA	DDM	0.002525316	83		R-III	TDM	0.002249998
39	[39]	GSK	DDM	0.000765347	84		PSO	TDM	0.002171714
40	[24]	GAMNU	DDM	0.000795540	85		CS	TDM	0.004569170
41	[18]	LCNMSE	DDM	0.000757590	86		ABC	TDM	0.002471743
42	[20]	EO	DDM	0.006348583	87		TLO	TDM	0.000779584
43	[21]	WHHO	DDM	0.000774553	88	[37]	ABC	TDM	0.000990246
44	[4]	CNMSMA	DDM	0.000757922	89		OBWOA	TDM	0.000823136
45	[5]	SMA	DDM	0.007025646	90		STLBO	TDM	0.000823698

**Table 3:** Comparison with the deterministic methods described in [63,64,65,66] for the RTC France solar cell using different methods for both SDM and ISDM.

Method	*I_pv_* (A)	*I*_01_ (μA)	*n*_1_	*R_P_* (Ω)	*R_SH_* (Ω)	*RMSE* Presented in [63]	*RMSE*
Laudani et al. [64]	0.7607884	0.3102482	1.4769641	0.03655304	52.859056	7.73009395 × 10^−4^	8.48634847817564 × 10^−4^
Cardenas et al. [65]	0.760788	0.3106847	1.4771051	0.036547	52.890468	7.730062729 × 10^−4^	8.48539429771994 × 10^−4^
TSLLS method [66]	0.76074014	0.31285196	1.4777295	0.036615485	55.907380	7.943924087 × 10^−4^	8.64560187331562 × 10^−4^
TSLLS with refinement [66]	0.76078797	0.31068485	1.4771052	0.036546942	52.889804	7.730062726 × 10^−4^	8.48514263124791 × 10^−4^

**Table 4:** Lower and upper bounds of the parameters and the results obtained for the Solarex MSX 60 module for both SDM and ISDM.

Parameter	Bounds		Model	
	Lower	Upper	SDM	ISDM
*I_pv_* (A)	3.7	4	3.81237	3.8110008
*I*_01_ (μA)	0.1	0.2	0.139907	0.13
*n*_1_	1.0	1.7	1.3325	1.3268061
*R_S_* (Ω)	0.1	0.5	0.22343	0.2255596
*R_P_* (Ω)	500	1500	897.00	940.0105
*R_SD_* (mΩ)	0.001	0.3	-	0.1047
*RMSE*			0.011705935	0.01167228

**Table 5:** *RMSE* calculation for Solarex MSX 60 module using various algorithms.

Method	Ref.	Algorithm	Model	*RMSE*
1	Proposed	SA-MRFO	ISDM	0.01167228000
2	Proposed	SA-MRFO	SDM	0.01170593500
3	[35]	ER-WCA	SDM	0.01170676846
4		HS	SDM	0.01286676449
5		COA	SDM	0.01198624631
6	[50]	NM	SDM	0.05563692890
7	[49]	BC	SDM	0.03072250565
8	[51]	A&I	SDM	0.01810661465
9	[52]	A&I	SDM	0.02839662736
10	[10]	CLSHADE	DDM	0.01202866517
11		CLSHADE	TDM	0.01165303676
12	[17]	TSO	TDM	0.01700978598

**Table 6:** Comparison of statistical results of different algorithms.

Parameters	PSO	COA [10]	MRFO	SA-MRFO
Mean	0.00194965	0.00086624	0.0009987	0.000781
Median	0.00078866	0.00077944	0.0007743	0.000773
Std	0.00315942	0.00039946	0.0004283	4.03 × 10^−5^

**Table 7:** *p*-values obtained with Wilcoxon’s rank-sum test (5% significance level).

Parameters	SA-MRFO vs. MRFO	SA-MRFO vs. COA	SA-MRFO vs. PSO
*p*-value	4.76 × 10^−5^	8.70 × 10^−5^	8.88 × 10^−7^

**Table 8:** Experimental results obtained from the experimentally tested solar modules.

Parameter/Model	SDM-Based COA [35]	SDM-Based SA-MRFO	ISDM-Based SA-MRFO
*R_S_* (Ω)	0.1140	0.1138	0.1142
*R_P_* (Ω)	219.75	222.05	250.53
*I*_0_ (A)	10.56 × 10^−8^	10.43 × 10^−8^	10.554 × 10^−8^
*I_pv_* (A)	0.2987	0.29868	0.2987
*n*	0.3441	0.3442	0.34405
*R_SD_* (mΩ)	-	-	0.41
*RMSE*	0.00113727	0.0011256	0.000912

## Data Availability

Not applicable.

## Abbreviations

ABC	Artificial bee colony
A&I	Analytical and iterative-based methods
BPFPA	Bee pollinator flower pollination algorithm
BBO	Biogeography-based optimization
BC	Bézier curves
BHCS	Biogeography-based heterogeneous cuckoo search
BLPSO	Biogeography-based learning particle swarm optimization
BPFPA	Bee pollinator flower pollination algorithm
BSA	Backtracking search algorithm
CLPSO	Comprehensive learning particle swarm optimization
CLSHADE	Chaotic LSHADE algorithm
CNMSMA	Chaotic Nelder–Mead slime mould algorithm
COA	Chaotic optimization approach
CPMPSO	Classified perturbation mutation-based particle swarm optimization
CWOA	Chaotic whale optimization algorithm
CSO	Cuckoo search optimization
DE	Differential evolution
DEBBO	Differential evolution Biogeography-based optimization
EHHO	Enhanced Harris Hawk Optimization
EO	Equilibrium Optimizer
EOTLBO	Either-or teaching learning-based algorithm
EAs	Evolutionary algorithms
EABOA	Enhanced adaptive butterfly optimization algorithm
ELPSO	Enhanced leader particle swarm optimization
ER-WCA	Evaporation-rate-based water cycle algorithm
FPSO	Flexible particle swarm optimization
FA	Firefly algorithm
FPA	Flower pollination algorithm
GA	Genetic algorithm
GAMS	General algebraic modeling system
GAMNU	Genetic Algorithm based on non-uniform mutation
GSK	Gaining–Sharing knowledge-based algorithm
GOTLBO	Generalized oppositional teaching learning-based optimization
HFAPS	Hybrid firefly and pattern search algorithms
HS	Harmony search
HCLPSO	Chaotic heterogeneous comprehensive learning particle swarm optimizer
ITLBO	Improved teaching–learning-based optimization
ISCA	Improved sine cosine algorithm
ISCE	Improved shuffled complex evolution
ILCOA	Improved Lozi map-based chaotic optimization algorithm
IJAYA	Improved JAYA optimization algorithm
JAYA	Sanskrit word meaning victory or triumph
LBSA	Learning backtracking search algorithm
LCNMSE	Laplacian Nelder–Mead spherical evolution
LETLBO	Teaching–learning-based optimization with learning experience
LMSA	Levenberg–Marquardt algorithm combined with simulated annealing
LSHADE	Successful history-based adaptive differential evolution variants linear population size reduction
MADE	Memetic adaptive differential evolution
MPSO	Modified particle swarm optimization
MSSO	Modified simplified swarm optimization algorithm
NM	Newton method
OBWOA	Opposition-based whale optimization algorithm
PSO	Particle swarm optimization
PGJAYA	Performance-guided JAYA algorithm
pSFS	Perturbed stochastic fractal search
R-II	Rao-2 algorithm
R-III	Rao-3 algorithm
SA	Simulated annealing
SATLBO	Self-adaptive teaching–learning-based optimization
SFS	Stochastic fractal search
SMA	Slime Mould Algorithm
STLBO	Simplified teaching–learning-based optimization
TLBO	Teaching–learning-based optimization
TLABC	Teaching–learning-based artificial bee colony
TSLLS	Two-step linear least-squares
TSO	Transient search optimization
WCA	Water cycle algorithm
WDO	Wind-driven optimization
WHHO	Whippy Harris Hawks optimization algorithm

## Appendix A

Table A1 shows the parameters of the solar RTC France cell using the methods presented in Table 2, while Table A2 shows the parameters of the Solarex MSX 60 module using the methods presented in Table 4.

**Table A1:** Parameters of the solar RTC France cell obtained using the literature methods presented in Table 2.

Method	Ref.	*I_pv_* (A)	*I*_01_ (μA)	*n*_1_	*R_S_* (Ω)	*R_P_* (Ω)	*I*_02_ (μA)	*n*_2_	*I*_03_ (μA)	*n*_3_
3	[18]	0.76077600	0.32302000	1.48118200	0.03637700	53.71822000	-	-	-	-
4	[20]	0.7607597037	0.32628893	1.48219300	0.03634099	54.20659400	-	-	-	-
5	[21]	0.76077551	0.32302031	1.48110808	0.03637710	53.71867407	-	-	-	-
6	[4]	0.76077600	0.32301700	1.48118200	0.03637700	53.71821000	-	-	-	-
7	[24]	0.76077400	0.32559540	1.48209600	0.03634020	53.89686000	-	-	-	-
8	[39]	0.76080000	0.32310000	1.48120000	0.03640000	53.72270000	-	-	-	-
9	[7]	0.760771077	0.32292900	1.481153457	0.036379593	53.76600144	-	-	-	-
10	[5]	0.76076000	0.32314000	1.48114000	0.03637000	53.71489000	-	-	-	-
11	[40]	0.76077553	0.32302083	1.48118359	0.03637709	53.71852514	-	-	-	-
12	[26]	0.76077500	0.32300000	1.48123800	0.03637500	53.74282000	-	-	-	-
13	[32]	0.76080000	0.32300000	1.48100000	0.03640000	53.71850000	-	-	-	-
14	[33]	0.76077000	0.32301000	1.48118000	0.03639000	53.71854000	-	-	-	-
15		0.76079000	0.32302000	1.48118000	0.03637000	53.71851000	-	-	-	-
16	[27]	0.76077600	0.32302100	1.48118400	0.03637700	53.71852000	-	-	-	-
17	[46]	0.76079000	0.31062000	1.47710000	0.03654800	52.88500000	-	-	-	-
18	[28]	0.76077552	0.32302000	1.48110817	0.03637000	53.71852000	-	-	-	-
19	[6]	0.76080000	0.32300000	1.48120000	0.03640000	53.71850000	-	-	-	-
20	[47]	0.76078000	0.32302000	1.48118000	0.03638000	53.71852000	-	-	-	-
21	[43]	0.76077562	0.32301700	1.48118220	0.03637716	53.71821748	-	-	-	-
22	[41]	0.76077500	0.32302100	1.48110800	0.03637700	53.71867900	-	-	-	-
23	[22]	0.76080000	0.32300000	1.48120000	0.03640000	53.71850000	-	-	-	-
24	[42]	0.76077450	0.32300180	1.48117740	0.03637750	53.73000000	-	-	-	-
25	[12]	0.76080000	0.32300000	1.48120000	0.03640000	53.71850000	-	-	-	-
26	[11]	0.76077600	0.32302000	1.48118400	0.03637700	53.71852400	-	-	-	-
27	[44]	0.76078000	0.32302000	1.48118000	0.03638000	53.71852000	-	-	-	-
28	[29]	0.76078700	0.31068300	1.47526200	0.03654600	52.88971000	-	-	-	-
29	[30]	0.76077700	0.32262200	1.48106000	0.03638190	53.67840000	-	-	-	-
30	[23]	0.76077553	0.32302083	1.48118360	0.03637709	53.71852771	-	-	-	-
31	[45]	0.76078000	0.32302000	1.48118000	0.03638000	53.71636000	-	-	-	-
32	[34]	0.76077600	0.32269900	1.48108000	0.03638100	53.69100000	-	-	-	-
33	[31]	0.76077700	0.32356400	1.48124400	0.03637000	53.74246500	-	-	-	-
34	[8]	0.76000000	0.31060000	1.47740000	0.03660000	57.71510000	-	-	-	-
35	[36]	0.76080000	0.32230000	1.48080000	0.03676800	57.74614000	-	-	-	-
36	[41]	0.76077000	0.32390000	1.48120000	0.03636000	53.79870000	-	-	-	-
37	[9]	0.76080000	0.32280000	1.48110000	0.03640000	53.75950000	-	-	-	-
38	[7]	0.76082865	0.25072000	1.45988481	0.03662660	55.36601290	0.720690	1.99997	-	-
39	[39]	0.76080000	0.25950000	1.46270000	0.03660000	54.93300000	0.479100	1.99830	-	-
40	[24]	0.76082700	0.32245246	1.48102800	0.03636440	53.11079000	0.000274	1.47010	-	-
41	[18]	0.76078100	0.74933000	2.00000000	0.03674000	55.48542000	0.225980	1.45102	-	-
42	[20]	0.76792000	0.39999000	2.00000000	0.03659000	54.17614000	0.266050	1.46451	-	-
43	[21]	0.76078094	0.22857400	1.45189500	0.03672887	55.42643282	0.727182	2.00000	-	-
44	[4]	0.76078100	0.22597600	1.45101700	0.036740	55.48545	0.750681	2.00000	-	-
45	[5]	0.76076000	0.74874000	2.00000000	0.03677	55.71456	0.226520	1.45463	-	-
46	[40]	0.76078108	0.22597468	1.45101692	0.03674043	55.48543568	0.749344	2.00000	-	-
47	[26]	0.760769017	0.58618400	1.968451449	0.036598831	55.63943956	0.240960	1.45691	-	-
48	[32]	0.76080	0.22730	1.45130	0.03670	55.43270	0.738400	1.99990	-	-
49	[33]	0.76078	0.74911	2.00000	0.03675	55.71854	0.226410	1.45471	-	-
50		0.76078	0.74814	2.00000	0.03674	55.71851	0.219110	1.45145	-	-
51	[42]	0.76078	0.22597	1.45102	0.03674	55.48542	0.749346	2.00000	-	-
52	[12]	0.76080	0.21031	1.44500	0.03680	55.81350	0.885340	2.00000	-	-
53		0.76080	0.13894	1.72540	0.03650	53.40580	0.262090	1.46580	-	-
54		0.76070	0.00608	1.84360	0.03640	52.65750	0.315070	1.47880	-	-
55		0.76080	0.23364	1.45380	0.03670	55.33820	0.684940	2.00000	-	-
56		0.76080	0.33673	1.48610	0.03610	55.06760	0.071730	1.93160	-	-
57		0.76070	0.25843	1.46250	0.03670	57.94220	0.386150	1.94350	-	-
58		0.76080	0.27189	1.46740	0.03660	61.13450	0.435050	1.96620	-	-
59		0.76060	0.00122	1.87910	0.03580	58.40180	0.372200	1.49560	-	-
60	[47]	0.76112	0.00237	1.68481	0.03619	52.40069	0.338750	1.48612	-	-
61		0.76056	0.17895	1.69574	0.03553	64.79937	0.315600	1.48789	-	-
62		0.76079	0.49461	1.88559	0.03671	54.65515	0.220690	1.45021	-	-
63		0.76078	0.65647	1.99990	0.03669	55.30604	0.237210	1.45509	-	-
64		0.76078	0.84161	2.00000	0.03679	55.72835	0.215450	1.44705	-	-
65	[30]	0.76101	0.00000	2.00000	0.03671	49.18670	0.292634	1.47134	-	-
66		0.76078	0.22597	1.45101	0.03674	55.48550	0.749358	2.00000	-	-
67		0.76081	0.19268	1.43800	0.03686	55.93352	0.999587	1.98372	-	-
58	[25]	0.76081	1.00000	1.83576	0.03755	55.92047	0.099168	1.38609	-	-
69		0.76162	0.41639	1.50537	0.03539	54.45518	0.000001	2.00000	-	-
70		0.76072	0.28670	1.46915	0.03666	58.29956	0.247485	1.96837	-	-
71		0.76886	0.66062	1.60874	0.02914	51.11600	0.455149	1.62890	-	-
72		0.76081	1.00000	1.83576	0.03755	55.92047	0.099168	1.38609	-	-
73	[48]	0.76078	0.25093	1.45982	0.03663	55.11700	0.545418	1.99941	-	-
74	[62]	0.76077	0.24150	1.45651	0.03666	55.20160	0.600000	1.98990	-	-
75	[9]	0.76010	0.00504	1.21860	0.03760	77.85190	0.750940	1.62470	-	-
76		0.76080	0.17390	1.65850	0.03650	54.30210	0.226640	1.45780	-	-
77		0.76070	0.24877	1.88170	0.03650	56.05240	0.274360	1.46820	-	-
78	[8]	0.76000	0.32110	1.47930	0.03640	59.62400	0.045280	2.00000	-	-
79	[21]	0.76078248	0.23910895	1.45393749	0.03672493	55.64995795	0.43972073	2.00000	0.8000	2.404159
80	[4]	0.760778	0.540044	1.996437	0.036636	55.09080	0.246533	1.45849	0.0143	1.785208
81	[5]	0.760785	0.2640	1.46085	0.03678	54.91521	0.00000551	1.14671	0.5810	2.020860
82	[33]	0.760792	0.2600	1.4608	0.03660	54.9149	0.000006	1.14660	0.5700	2.020800
83		0.760791	0.2100	1.7714	0.03670	55.3571	0.220000	1.45130	0.9900	2.410300
84		0.760782	0.2500	1.4601	0.03660	55.3133	0.041000	1.74090	1.0000	2.251400
85		0.760776	0.1400	1.4872	0.03630	53.7218	0.190000	1.47710	0.0310	4.466300
86		0.760790	0.3200	1.8666	0.03670	55.4411	0.230000	1.45210	0.7400	2.394900
87		0.760763	0.2800	1.4684	0.03650	55.3821	0.000670	1.54680	1.0000	2.322500
88	[37]	0.760700	0.2000	1.4414	0.03687	55.8344	0.500000	1.90000	0.2100	2.000000
89		0.760770	0.2353	1.4543	0.03668	55.4448	0.221300	2.00000	0.4573	2.000000
90		0.760800	0.2349	1.4541	0.0367	55.2641	0.229700	2.00000	0.4443	2.000000

**Table A2:** Parameters of the Solarex MSX 60 module obtained using the literature methods presented in Table 4.

Method	Ref.	Method	*I_pv_* (A)	*I*_01_ (A)	*n*_1_	*R_S_* (Ω)	*R_P_* (Ω)	*I*_02_ (A)	*n*_2_	*I*_03_ (A)	*n*_3_
1	[35]	ER-WCA	3.8121	0.1399 × 10^−6^	1.3325	0.22351	914.6885	-	-	-	-
2		HS	3.8115	0.2265 × 10^−6^	1.3707	0.21287	1976.07	-	-	-	-
3		COA	3.81	0.1783 × 10^−6^	1.3514	0.21840	2004.977	-	-	-	-
4	[50]	NM	3.8084	4.8723 × 10^−10^	1.0003	0.3692	169.0471	-	-	-	-
5	[49]	BC	3.808	1.22 × 10^−9^	1.045	0.316	146.08	-	-	-	-
6	[51]	A&I	3.7983	6.79 × 10^−8^	1.28	0.251	582.7278	-	-	-	-
7	[52]	A&I	3.808244	1.21946 × 10^−9^	1.045334	0.316000	146.081207	-	-	-	-
8	[10]	CLSHADE	3.812527	0.12311 × 10^−6^	1.32290	0.226800	800	7.2999 × 10^−11^	1.9880	-	-
9		CLSHADE	3.81253	0.12311 × 10^−6^	1.32270	0.226760	823.40000	7.29985 × 10^−11^	1.9900	1.25 × 10^−10^	1.93000
10	[17]	TSO	3.8019	3.3525 × 10^−7^	1.9346	0.22724	450.13	1 × 10^−12^	1.7208	6.457 × 10^−8^	1.2764
